# Effectiveness of current psychological interventions to improve emotion regulation in youth: a meta-analysis

**DOI:** 10.1007/s00787-020-01498-4

**Published:** 2020-02-27

**Authors:** Bettina Moltrecht, Jessica Deighton, Praveetha Patalay, Julian Edbrooke-Childs

**Affiliations:** 1 Division of Psychology and Language Sciences, University College London, 26 Bedford Way, Bloomsbury, London, WC1H 0AP UK; 2 Anna Freud National Centre for Children and Families, London, UK; 3 Centre for Longitudinal Studies and MRC Unit for Lifelong Health and Ageing, University College London, London, UK

**Keywords:** Emotion regulation, Psychopathology, Youth mental health, Meta-analysis, Psychological intervention

## Abstract

Research investigating the role of emotion regulation (ER) in the development and treatment of psychopathology has increased in recent years. Evidence suggests that an increased focus on ER in treatment can improve existing interventions. Most ER research has neglected young people, therefore the present meta-analysis summarizes the evidence for existing psychosocial intervention and their effectiveness to improve ER in youth. A systematic review and meta-analysis was conducted according to the PRISMA guidelines. Twenty-one randomized-control-trials (RCTs) assessed changes in ER following a psychological intervention in youth exhibiting various psychopathological symptoms. We found moderate effect sizes for current interventions to decrease emotion dysregulation in youth (*g* = − 0.46) and small effect sizes to improve emotion regulation (*g* = 0.36). Significant differences between studies including intervention components, ER measures and populations studied resulted in large heterogeneity. This is the first meta-analysis that summarizes the effectiveness for existing interventions to improve ER in youth. The results suggest that interventions can enhance ER in youth, and that these improvements correlate with improvements in psychopathology. More RCTs including larger sample sizes, different age groups and psychopathologies are needed to increase our understanding of what works for who and when.

**Electronic supplementary material:**

The online version of this article (10.1007/s00787-020-01498-4) contains supplementary material, which is available to authorized users.

## Introduction

Most common mental health disorders, including depression, substance abuse, eating disorders and anxiety have their onset during adolescence [1]. It has been argued that this peak in psychopathological symptoms results from developmental changes, which hamper emotion regulation (ER) [2]. ER has been broadly defined as “the extrinsic and intrinsic processes responsible for monitoring, evaluating, and modifying emotional reactions, especially their intensive and temporal features, to accomplish one’s goals” [3]. These regulatory processes comprise physiological, experiential, behavioural, as well as psychological components [4]

The concept of ER has faced significant definitional challenges in the past decades, with hundreds of research papers referring to it each year in various direct and indirect ways, but the majority do not provide a clear definition. One of the most influential definitions has been presented by James Gross, who introduced the *Process* *model* *of* *emotion* *regulation*, according to which we are able to modify emotional experiences at different points throughout the ER process by implementing different ER strategies [4, 5]. Gross clustered the ER strategies into categories based on the point in time at which they are applied during the ER process: *situation* *selection* (e.g. “I am worried that I will do badly on the test today, so I might rather not go”), *selection* *modification* (e.g., “My mom dropped me off at school, although I wasn’t feeling well. I could *turn* *around* or perhaps, I can *ask* my friend Johnny *for* *help* before the test), *attentional* *deployment* to certain aspects of the situation (e.g., “I am so nervous, I can *hear* my heart racing. I will try *distracting* myself with some music”), *cognitively* *changing* the meaning of a situation (e.g.,” It would be bad if I failed this test today. Luckily there is another test in 4 weeks”), and finally *modifying* *the* *response* to the emotion eliciting event (e.g., “The test was a *catastrophe”.* I *told* my mom about it and *cried.* I was so sad. She *gave* me a *hug* and said: “We cannot change what happened, but we can prepare better for the next test”).

Past research has identified various ER strategies for each of the above stages. Frequently, researchers have attempted to divide them into maladaptive (e.g., catastrophizing, rumination, avoidance, suppression) or adaptive (e.g., problem-solving, acceptance, savouring, cognitive reappraisal) strategies depending on their assumed impact on psychopathological symptoms.

### Emotion regulation and psychopathology

One of the most comprehensive systematic reviews by Aldao et al. [6] looked at the relationship between six different ER strategies and four different psychopathologies, including depression, anxiety, eating disorders and substance abuse. The authors found that the six strategies, avoidance, problem-solving, reappraisal, suppression, rumination and acceptance, were all associated with the different types of psychopathology. More specifically, they found that avoidance and suppression were positively associated with anxiety, depression and eating disorders, while rumination was positively associated with anxiety, depression, eating- and substance-abuse disorders. Problem-solving and reappraisal correlated negatively with psychopathological symptoms, while acceptance showed no significant association with depressive or anxiety symptoms. Further moderator analyses demonstrated that age (child vs. adult) significantly moderated the association between suppression, problem-solving, and depression, with adults showing significantly larger effect sizes than children. Age group was however not a significant moderator for the links between rumination and depression.

Aldao’s systematic review results were primarily based on data derived from adult studies, with only six of the 114 studies including data on children or adolescent samples. However, similar findings demonstrating the close association between emotion dysregulation and psychopathology have also been reported for studies focusing on young populations. Schäfer et al. [7] summarized the evidence for different ER strategies in youth exhibiting sub-clinical symptoms of anxiety and depression. Similarly, they found that depression and anxiety had the strongest positive association with avoidance and rumination; but the strongest negative association with acceptance. Their review focused on adolescents in the ages of 13–18 years with sub-clinical symptoms, therefore no conclusions could be made regarding younger groups or those displaying severe clinical symptoms. Evidence from studies looking at other youth mental disorders such as attention-deficit/hyperactivity disorder, conduct disorder, eating disorders and borderline-personality disorder have reported similar patterns [8–11].

Most research looking at ER in clinical populations has focused on emotion dysregulation and strategies to regulate negative emotions, such as sadness or anger; while ER as an ability or positive ER strategies (e.g. savouring or gratitude) have been widely neglected [12]. Hence in the present review the term emotion dysregulation will refer to having difficulties, while the term emotion regulation will refer to abilities or skills. Furthermore, it will include both, strategies to regulate positive as well as negative emotions. Additionally, Aldao et al. [13, 14] highlighted that effective ER does not come down to mere down regulation of negative emotions and upregulation of positive emotions, but whether the individual is able to flexibly apply strategies that match the respective situation. Hence the present systematic review will also include ER measures that assess flexible ER.

Despite the growing evidence highlighting the importance of ER in the development of youth psychopathology, it remains unclear whether ER difficulties are a risk factor or a consequence of psychopathology. Until now, only a few studies have shed light on the nature of this relationship. McLaughlin et al. [15] investigated emotion dysregulation patterns in adolescents exhibiting different psychopathological symptoms (i.e. depression, anxiety, aggression, eating pathology) before and after a seven month period. They found that emotion dysregulation (in their study a latent factor based on low emotional understanding, emotion expression and ruminative response to distress), predicted increased symptoms seven months later for all psychopathologies, but depression. Emotion dysregulation on the other hand was not predicted by earlier psychopathological symptoms. Due to the limited availability of longitudinal studies so far, another way to explore this relationship would be through intervention studies that include mediation analyses, based on which one could conclude whether changes in ER lead to changes in psychopathology.

### Emotion regulation in interventions

Due to findings relating ER to a wide range of mental disorders, ER has been argued to represent a transdiagnostic core feature underlying these disorders [16]. Transdiagnostic frameworks propose that multiple mental disorders are caused and maintained by a similar subset of underlying processes, which finds further support in high comorbidity rates among the disorders and observations showing that different disorders respond to similar treatments. Following this, it has been suggested that psychological interventions can be improved by having an increased focus on ER [17], as it is the case in most third wave interventions including mindfulness-based cognitive therapy (MBCT), dialectical-behavioural therapy (DBT) or acceptance-based behavioural therapy (ACT). Evidence from the adult literature indicates that promising psychological interventions did improve ER, and that these improvements mediated decreases in psychopathological symptoms [18]. Sloan et al. [19] recently examined whether changes in ER related to symptom reduction in anxiety, depression, substance abuse, eating and borderline personality disorder. They found that the use of maladaptive ER strategies improved following treatment, regardless the type of intervention or disorder. However, their systematic review only included participants older than 13 years.

### Objective

The present meta-analysis aims to summarize the effectiveness of psychological interventions to improve ER in youth. To our knowledge there is no meta-analysis that has looked at research involving youth samples. Moreover, it focuses on emotion dysregulation, related strategies as well as ER abilities and related strategies. Finally, mediation analyses of changes in ER and psychopathology in response to interventions will be summarized.

We aim to answers the following research questions:

- Do existing psychological interventions effectively improve emotion (dys-) regulation in youth?
- Are improvements in emotion (dys-) regulation associated with changes in psychopathological symptoms?

## Methods

### Literature search

We followed the PRISMA guidelines for the present systematic review [20]. The literature search of the electronic databases was conducted on the 4th of December, 2017 and updated on the 9th of April, 2018 using the following electronic databases: Ovid/Medline, Ovid PsychINFO and Web of Science (a detailed overview of the search strategy can be found in the supplementary materials). Identified publications were downloaded from the databases and saved to a reference manager on the dates specified above. If relevant literature reviews were identified during the abstract screening process (see below), we manually screened their reference lists for further important publications. Our literature search was restricted to peer-reviewed journal articles written in English. Peer-reviewed publications have been assumed to increase the inclusion of studies with higher research quality.

### Inclusion and exclusion criteria

#### Inclusion criteria

- Children and adolescents between the ages of 6–24 years. Research with younger children was excluded because it primarily involves observational methods. In line with recent definitions of “adolescence” we included the age of 24 [21].
- Sample with depression, anxiety, eating disorder, substance abuse, attention-deficit hyperactivity disorder, borderline personality disorder symptom, as these have been shown to share common ER difficulties [6, 7, 19].
- Intervention aims to improve ER and symptoms relating to any of the mental health disorders mentioned above.
- Randomized and quasi-randomized control studies
- Any control condition
- Self-, parent, teacher or professional report through validated ER measure

#### Exclusion criteria

- Adult population
- Symptoms not relating to disorders mentioned above
- No measure of emotion regulation included
- Special populations (e.g. autism spectrum disorder, intellectual impairment, medical condition)
- Medical or pharmacological intervention
- No manual or description of intervention and the assumed active component
- No control group present
- Studies reporting outcomes of neural correlates only (e.g., fMRI)

### Study selection

All identified articles were added to a systematic review software (Eppi-Reviewer). Duplicates were removed and abstracts and titles were screened based on the inclusion and exclusion criteria. The method section of each paper was screened for valid ER measures. All studies with a valid ER measure entered the full-text screening stage. A second researcher (D.M) randomly reviewed and rated 25% of the selected title and abstract papers. Where there was a disagreement (4%) regarding the inclusion of a study, the two researchers reviewed the article and discussed its eligibility until an agreement was achieved.

### Data extraction

Information relating to study characteristics including: authors, year of publication, study design, intervention type, definition and measurement of ER, comparison group, study results (including sample size, age group, participation rate, attrition, relevant clinical and ER outcomes) and information to determine any study bias was extracted from each study. Correlations between changes in ER and clinical outcomes were collected if reported. Coding options for categorical variables are provided in the supplementary materials.

### Outcome measures

#### Emotion regulation and dysregulation

Studies with any validated self-report measure to assess ER difficulties or skills, either as a single factor or in terms of the ER strategies, were included (see supplementary materials for an overview of included measures). We used Adrian et al. [22] review of emotion regulation assessment and similar reviews [6, 19] as guidance to decide on a measures’ eligibility. The authors of the present review acknowledge that some ER measures may have substantial overlap with measures assessing psychopathological symptoms, which are addressed in more detail in the discussion. The two meta-analyses included (a) studies that assessed emotion dysregulation (i.e., lack of access to strategies, difficulties accepting negative emotions) or any of the associated maladaptive ER strategies including: avoidance, suppression, catastrophizing, rumination and (b) ER ability (e.g., ER flexibility, emotional understanding) and any of the associated adaptive ER strategies including: acceptance, savouring, gratitude, cognitive reappraisal, problem solving and mindfulness (a complete list is provided in the supplements). We extracted all available data reported for subscales and overall mean scores. If possible we calculated overall mean scores, based on the subscales data provided. For the meta-analyses, all available effect sizes (subscale or full scale) were combined according to their categorization into emotion regulation or dysregulation (see supplements for coding scheme).

#### Psychopathology

Is treated as a secondary outcome measures in the present review as it was only used to answer our second research question, regarding the association between change in ER and change in psychopathology in response to treatment. Psychopathology symptoms were either based on self-report measures or clinician ratings (e.g., Beck Depression Inventory). If a study reported more than one scale for the same disorder category, we chose one measure based on its reliability and whether it had been used in one of the other studies in the present review. Reported mean scores were used to calculate standardised effect sizes, which were then entered in the meta-regression analysis.

### Quality and risk of bias assessment

Two researchers (BM and DM) independently assessed the methodological quality of the included studies (interrater agreement = 98%) using the Effective Public Health Practice Project Quality Assessment tool (EPHPP). The EPHPP evaluates the quality of each study based on their rating, ranging from strong, moderate to weak, across the following six categories: selection bias, study design, the presence of confounding variables, blinding, data collection methods, and participant withdrawals and drop-outs. The EPHPP has been reported suitable for systematic reviews and evidence has shown good content and construct validity [23, 24].

### Data analysis

A primary analysis was conducted to detect any influential studies in the data-set. This was done through the “metaninf” command in Stata, which indicates each study’s impact on the overall effect size if that study is omitted from the analysis. Furthermore, we assessed each studies level of heterogeneity through a Galbraith plot (“galbr” command in Stata) [25]. Studies with a great impact on the overall effect size and larger than expected level of heterogeneity, were regarded as influential studies. Subsequent meta-analyses were conducted with and without these studies, in order to identify their respective impact on the results. In line with current recommendations for meta-analysis models in psychology, we conducted two random effects models: one with emotion dysregulation as a primary outcome and one with ER abilities as the primary outcome [26]. To explore sources of heterogeneity we conducted a series of sub-group analyses. Subgroup analyses help identify whether there are differences in effect size or heterogeneity due to study-level factors (see “Meta-regression and subgroup-analyses” for more detail below). Furthermore, we conducted a meta-regression with effect size as the dependent variable and intervention type, age group, control group and quality rating as the predictor variables. A combination of these two approaches has been recommended [27]. In order to answer the second research question we conducted a second meta-regression, with effect sizes of psychopathological symptoms as the dependent variable, and effects sizes of improved ER as the predictor variable.

### Effect sizes

Treatment effect was estimated using the weighted mean effect size Hedges’ *g*. Hedges’ *g* is interpreted like Cohen’s *d*, with effect sizes ranging from small (0.2), medium (0.5) to large (0.8) [28]. Hedges’*g* (see Formula 1) and the standard error were calculated based on standardized mean-differences, standard deviations and sample sizes. This data was entered into Stata and the “meta” command was used to conduct the random effects models.

1 $$g_{{{\text{hedges}}}} = \frac{{M_{1} - M_{2} }}{{s_{{{\text{pooled}}}} }}\;{\text{with}}\;s_{{{\text{pooled}}}} = \sqrt {\frac{{(n_{1} - 1)s_{1}^{2} + (n_{2} - 1)s_{2}^{2} }}{{n_{1} + n_{2} - 2}}} .$$

Formula 1—Hedges’ *g*.

For studies with multiple treatment groups, the decision on how to include them, was made on a case-by-case basis with regards to the research question. In accordance with the Cochrane handbook the following options were considered [29]:

- One of the treatment conditions was excluded if the treatment’s main target was not ER or any related concept and did therefore not add any additional insight to the research question.
- Effect sizes of two treatment groups were pooled and compared to the control group, if the intervention groups were similar enough to be combined.
- Each treatment group was entered as a single comparison group, by splitting the control group in half, if combining or excluding one condition would have resulted in loss of information. This approach was adopted where both interventions were assumed to improve ER, but differences between the conditions added valuable insights, e.g., whether one intervention could be more effective than the other.

### Heterogeneity

Heterogeneity between the studies was assessed with the *Q* statistic, *I*^2^ and *T*^*2*^. The *Q* test follows the chi-square distribution and estimates the probability of sampling error being the only cause for variance. A significant *Q* test indicates that heterogeneity is present. However, it does not provide sufficient information about the source of heterogeneity. Therefore, *I*^2^ and *T*^2^ were also taken into account. *T*^2^ describes the between-study variance, while *I*^2^ describes what proportion of the observed variance in the effect estimates is due to systematic differences between the studies rather than sampling error. Smaller values of *I*^2^ suggest that the observed heterogeneity is mostly random, while larger values suggest study-level differences. The following levels of heterogeneity have been identified for *I*^2^: low: *I*^2^ = 25%, medium: *I*^2^ = 50%, and high: *I*^2^ = 75% [30]. We also calculated 95% prediction intervals (PI; see Formula 2) [31], which aim to predict the range of possible population parameters in future empirical studies (e.g., we expect that in future studies 95% of the true effects lie within this interval). Hence, PI’s are different from confidence intervals, which estimate the precision of the mean effect size in the general population.

2 $$\hat{\mu } - t_{k - 2} \sqrt {\hat{\tau }^{2} + {\text{SE(}}\hat{\mu }{)}^{{2}} } ,\;\hat{\mu } + t_{k - 2} \sqrt {\hat{\tau }^{2} + {\text{SE(}}\hat{\mu }{)}^{{2}} } .$$

Formula 2—prediction interval

### Meta-regression and subgroup analyses

A meta-regression was performed to identify possible moderating effects of certain between study-level characteristics. The meta-regression was conducted with the “meta regress” command in Stata 16. Categorical variables are automatically dummy-coded by the software and the resulting estimates indicate how the effect size of each subgroup differs with respect to the chosen reference group. Furthermore, separate subgroup analyses with each relevant moderator were conducted to explore potential sources of heterogeneity and their impact on the overall effect size. With respect to the present research question the following subgroup analyses were conducted:

- *Type* *of* *intervention*: distinguished between two types of interventions, those with a specific focus on ER (e.g., emotion focused CBT, emotion regulation training, or any of the third wave interventions) and non-specific interventions (e.g., standard CBT, motivational interviewing). An intervention was coded as ER specific, if they included specific ER modules or tasks; or if these were stated to take up most of the content or time, compared to other modules in the intervention programme. (See Table 5 in supplements for intervention descriptions).
- *Type* *of* *control* *group*: compared studies with active versus passive control groups. Passive control groups included studies with a waitlist or assessment-only design, while active control groups included any type of intervention, including treatment as usual.
- *Type* *of* *emotion* *regulation* *strategy*: compared studies based on different types of ER strategies. Subgroups could only be formed if sufficient data was available. (See supplements for specific ER strategies).
- *Type* *of* *disorder*: compared effectiveness of studies relating to different types of disorders. Studies were categorized based on the authors’ description of the recruited sample and the diagnostic tools employed. Six main categories were included: (a) anxiety disorders, including generalized anxiety, phobias, PTSD, obsessive compulsive disorder; (b) depression, including major depressive disorder, bipolar disorder, suicidal thoughts; (c) ADHD; (d) borderline personality disorder; (e) substance abuse (f) eating disorders.
- *Age* *groups*: differences in effectiveness for different age groups was explored by creating a new categorical variable for age with four levels. Studies with a participant mean age under 10 years, were categorised as “child” population. “Early adolescence” included samples with a mean age between 10 and 13 years. Studies with participants older than 13 years, but younger than 17 were categorised as “adolescence”. The fourth category “late adolescence” included all samples with a mean age larger than 17 years but younger than 25 years.
- *Quality* *of* *study*: to investigate whether there was a difference in effect size depending on quality ratings. Studies were rated as being of low (3), moderate (2) or high (1) quality.

### Publication bias

Publication bias was visually assessed with the help of a funnel plot. No publication bias was assumed if the points in the scatter plot form the shape of a funnel, while an asymmetrical shape suggests a publication bias. Furthermore, the Egger’s test was applied to test for small-study effects whereby precision seems to be related to the effect size estimate. Fail-safe *N* statistics were not performed due to unreliability [32]

### Relationship between ER and psychopathological symptoms

To assess whether improvements in psychopathological symptoms were associated with changes in ER, a meta-regression was conducted, with effect sizes of psychopathological symptoms as the dependent variable, and effects sizes of improved ER as the predictor variable.

## Results

### Study selection

**Fig. 1:**
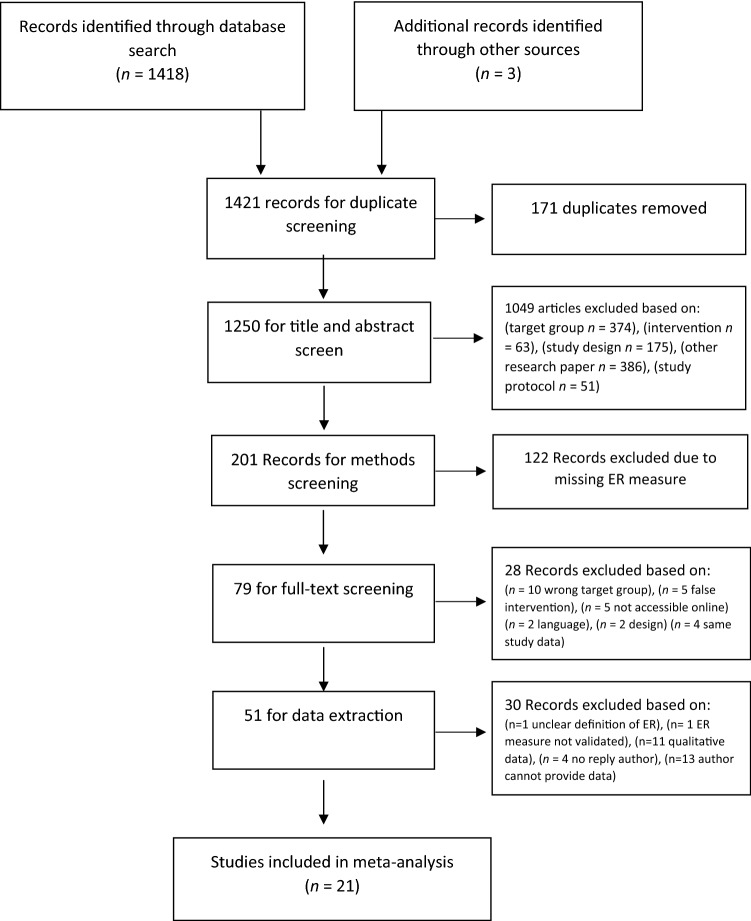
Flow diagram

The search identified 1418 articles. After duplicates (*n* = 171) were removed 1250 papers were included for the abstract and title screening. 1049 articles were excluded based on the abstract and title screening. Of the remaining 201 papers, 122 papers had to be excluded due to missing ER measures. In total, 79 studies entered the full-text screening, of which 34 studies matched the selection criteria and provided sufficient data. Another 17 studies, matched the criteria, but the authors had to be contacted to provide additional information that could not be derived from the published article. During the data extraction phase 30 studies were excluded. Four of those were excluded because the authors were not accessible [33–36]. Finally, 21 independent studies were included in the meta-analysis, from which 33 treatment effects were extracted (19 emotion dysregulation, 14 emotion regulation; see Fig. 1 for study selection process).

### General study characteristics

The characteristics of the included studies are summarized in Table 1. For studies with multiple treatment conditions, where both treatment conditions were assumed to have an effect on ER, both groups were included in the analysis, by splitting the control group in half and pairing it with each treatment group. For the remaining studies (*k* = 3), the second treatment group was excluded. All of the included studies showed a large variety regarding the type of ER measure and intervention employed a detailed description of these is provided in the supplementary materials, see Table 5. CBT was the most commonly employed intervention (*k* = 16) and almost all interventions included some kind of CBT components. Eight studies stated to specifically address emotion dysregulation (i.e. emotion regulation training). Four interventions targeted specific ER strategies (i.e., rumination or mindfulness; see Table 1).

### Quality and publication bias

Quality ratings for each study are shown in Table 1. All studies were randomized control studies, however nine studies reported baseline differences between the groups, while two studies did not provide any information on potential baseline differences. One study did not provide any information about the control condition, six studies compared the intervention with a treatment as usual condition.

**Table 1:** Study characteristics

Study	Psychopathology	Design	*N*	Age	Conditions	ER measure	Quality rating
Slee et al. (2008)—The Netherlands	BPD	RCT	82	24.2	CBT − TAU	DERS	Strong
Schuppert et al. (2012)—The Netherlands	BPD	RCT	109	15.98	ERT − TAU	LPI subscale—emotion dysregulation	Strong
Suveg et al. (2017)—USA	AD	RCT	92	8.93	ECBT − CBT	ERC	Moderate
Dingle et al. (2017)—Australia	AD, MD	RCT	51	18.68	ERP − WL	DERS	Moderate
Hides et al. (2011)—Australia	MD, SUB	RCT	88	19.2	CBT + MI – TAU	CISS	Weak
Atkinson et al. (2016)—Australia	ED	RCT	33	20.57	MF − WL Dissociation^a^	FFMQ	Weak
Azrin et al. (2001)—USA	CD, SUB	RCT	56	15.4	CPS − FBT	SPSI-R	Moderate
Stasiak et al. (2014)—New Zealand	MD	RCT (pilot)	34	15.2	cCBT − TAU	ACS-PS	Moderate
Jacobs et al. (2016)	MD	RCT	33	15.5	RCBT − WL	RRS	Strong
Livheim et al. (2015)—Australia	MD	QRCT	51	14.6	ACT − TAU	AFQ	Strong
^1^Livheim et al. (2015)—Sweden	MD	RCT	32	14.5	ACT − TAU	AFQ, MAAS	Weak
Kaufman et al. (2005)—USA	MD, CD	RCT	93	15.1	CBT − LS	IC-PS	Strong
Hennesdottir et al. (2017)—Iceland	ADHD	RCT (pilot)	30	9.2	CBT − WL Parent training^a^	ERC	Strong
Meisner-Stedman et al. (2017)	AD	RCT	29	24.56	CTPTSD − WL	Rumination items	Strong
^1^Essau et al. (2012)—Germany	AD	CRCT	638	10.91	CBT − WL	CSCY-PS	Moderate
Latimer et al. (2003)—USA	SUB	RCT (pilot)	43	16.07	CBT − DHPE	SPSI	Moderate
Winters et al. (2012)	SUB	QRCT	192	16.13	MI-A − WL MI-P^a^	PSQ	Moderate
Smith et al. (2015)	MD	RCT	109	13–16	cCBT − WL	CRSQ	Strong
Fitzpatrick et al. (2005)—USA	MD	RCT	94	19.02	PS − Health Education	SPSI-R	Moderate
Multi-treatment trials entered with split groups							
Hancock et al. (2016)—Australia	AD	RCT	99	13.8	ACT − WL	AFQ	Strong
	AD	RCT	94	13.8	CBT − WL	AFQ	Strong
Afshari et al. (2014)—Iran	AD	RCT	77	10.57	ERT− WL	CERQ, CEMS	Weak
	AD	RCT	55	10.57	CBT− WL	CERQ, CEMS	Weak

*BPD* borderline personality disorder, *AD* anxiety disorder, *MD* major depression, *SUB* substance abuse, *ED* eating disorder, *CD* conduct disorder, *ADHD* attention deficit hyperactivity disorder, *RCT* randomized control trial, *QRCT* quasi-randomized control trial, *CBT* cognitive behavioural therapy, *TAU* treatment as usual, *ERT* emotion regulation training, *ECBT* emotion-focussed CBT, *ERP* emotion regulation program, *WL* waitlist, *MI* motivational interviewing, *MF* mindfulness, *CPS* cognitive problem solving, *FBT* family behavioural therapy, *cCBT* computerized CBT, *RCBT* rumination focussed CBT, *ACT* acceptance and commitment therapy, *LS* life skills, *CT-PTSD* cognitive therapy for PTSD, *DGPE* drugs harm psychoeducation curriculum, *MIA* motivation interviewing adolescence, *MI-P* motivation interviewing parents, *PS* problem solving, *DERS* difficulties with emotion regulation scale, *LPI* life problems inventory, *ERC* emotion regulation checklist, *CISS* coping inventory for stressful situations, *FFMQ* five factor mindfulness questionnaire, *SPSI-R* social problem solving inventory-revised, *ACS-PS* adolescent coping scale-problem solving, *RRS* ruminative response scale, *AFQ* avoidance and fusion questionnaire, *MAAS* mindful attention awareness scale, *IC-PS* issues checklist-problem solving, *CSCY* coping scale for children and youth, *PSQ* problem solving questionnaire, *CRSQ* child response style questionnaire, *CERQ* cognitive emotion regulation questionnaire, *CEMS* children’s emotion management scale

^a^Condition was part of multi-treatment trial and was excluded from meta-analysis ^1^outlier study removed from main analysis

### Meta-analysis: effectiveness of interventions to reduce emotion dysregulation

The first random effects model was based on the original 19 effect sizes from 17 independent studies, which indicated a medium treatment effect (*g* = 0.52), 95% CI [− 0.86, − 0.18], *p* < 0.001). Due to large heterogeneity *I*^2^ = 90.87% (*Q* = 129.64, *df* = 18, *p* < 0.001), we decided to run the “metainf” command and a Galbraith plot to identify highly influential studies [25, 37]. The results (see Plots 1 and 2 in supplementary materials) indicated that two studies, one by Slee et al. [38] and one of Livheim and colleague’s studies (based in Sweden [39]) had a significant impact on the overall effect size, while also contributing to a large amount of heterogeneity. We regarded these studies as highly influential studies and removed them from the main model, which effectively decreased the level of heterogeneity by *I*^2^ = 18.05% [37]. (Results of the full and the reduced meta-analysis model are presented in Table 2 and supplementary materials). Results of the reduced model are discussed in more detail below.

**Table 2:** Random effect models and sub-group analyses with emotion dysregulation as outcome

Reduced data set	*m*	*k*	*n*	Hedges *g*	95% CI	*p* (*z* test)	*Q*	*p* (Q)	*T*^2^	*I*^2^ (%)
Emotion dysregulation	17	15	1744	− 0.46	− 0.67, − 0.26	0.00	54.06	0.00	0.12	72.82
Emotion dysregulation by										
Intervention										
CBT intervention	8	8	1058	− 0.40	− 0.64,− 0.15		14.84	0.02	0.06	59.37
ER intervention	7	7	598	− 0.51	− 0.82,− 0.20		23.34	0.00	0.46	70.38
Control group										
Active control	8	8	532	− 0.19	− 0.41, − 0.03		10.44	0.11	0.03	39.45
Passive control	9	7	1212	− 0.66	− 0.93, − 0.39		39.33	0.00	0.12	71.47
Quality rating										
Strong	9	8	612	− 0.59	− 0.85, − 0.33		16.35	0.02	0.08	57.22
Moderate	5	5	973	− 0.13	− 0.26, − 0.01		5.95	0.31	0.00	0.0
Weak	3	2	159	− 0.81	− 1.40, − 0.22		6.38	0.04	0.18	66.58
Full-data set										
Emotion dysregulation	19	16	1851	− 0.52	− 0.86, − 0.18	0.00	129.64	0.00	0.49	90.87
Emotion dysregulation by										
Intervention										
CBT intervention	8	8	1143	− 0.71	− 1.26,− 0.15		73.69	0.00	0.58	93.20
ER intervention	9	8	623	− 0.35	− 0.80, 0.10		44.07	0.00	0.40	86.54
Control group										
Active control	8	8	639	− 0.32	− 0.99, − 0.35		90.31	0.00	0.98	94.03
Passive control	9	7	1212	− 0.66	− 0.92, − 0.39		38.31	0.00	0.12	70.89
Quality rating										
Strong	9	8	691	− 0.86	− 1.35, − 0.36		61.08	0.00	0.50	89.23
Moderate	6	6	973	− 0.13	− 0.26, − 0.01		5.74	0.33	0.00	0.0
Weak	4	3	187	− 0.81	− 1.37, − 0.84		33.81	0.02	1.16	91.87

**Fig. 2:**
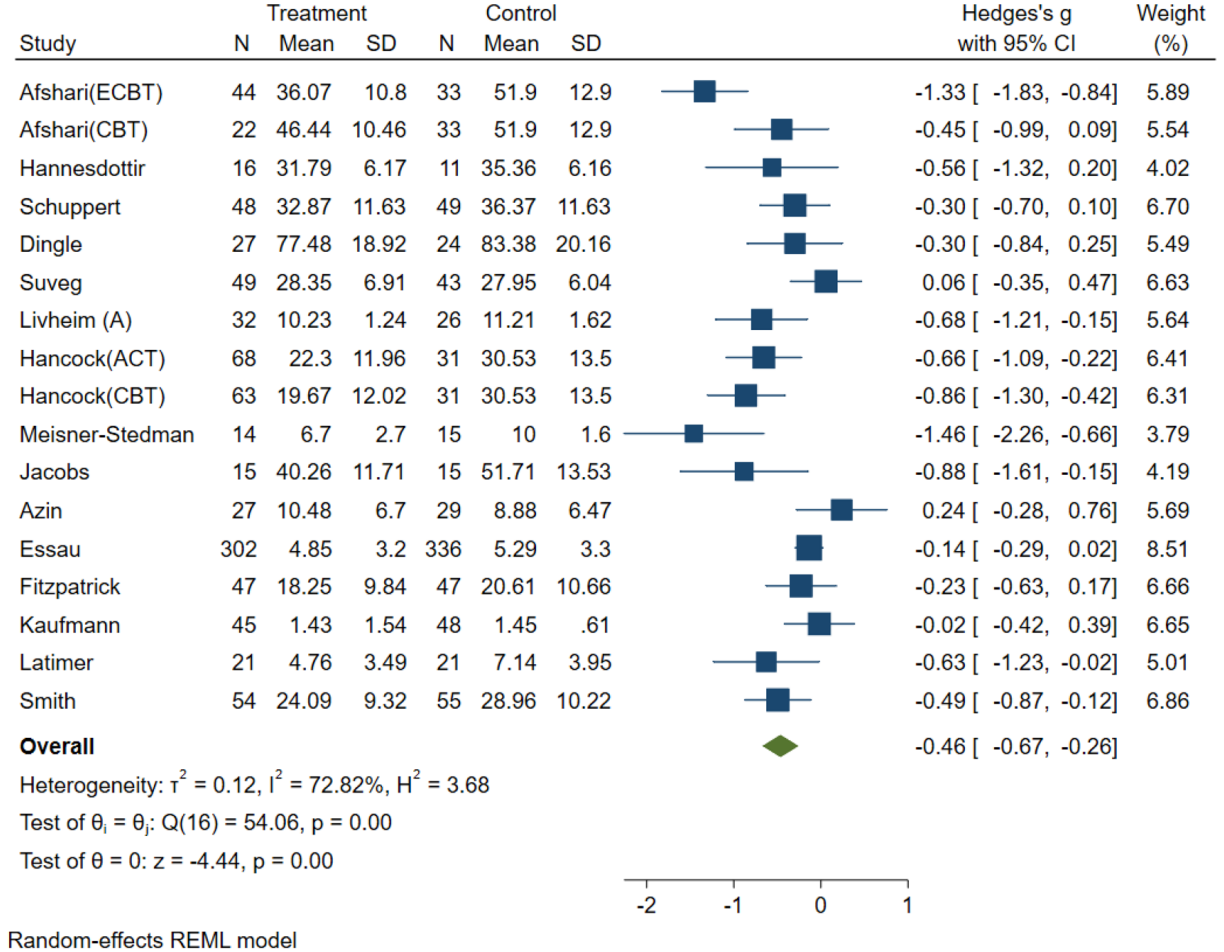
Forest plot: random-effects model (reduced) with emotion dysregulation as primary outcome

The forest plot and confidence intervals (CI) show that eight studies significantly reduced emotion dysregulation (CIs are entirely on the negative side), while the remaining studies (*k* = 9) showed no significant treatment effect (Fig. 2). Overall, the results indicate a medium treatment effect (*g* = − 0.46), 95% CI [− 0.67, − 0.26], *p* < 0.001). The confidence interval (no value of 0 is present), and the *z* statistic (*z* = − 4.44, *p* < 0.001) suggest that the null hypothesis (H_0_: intervention had no impact on emotion dysregulation) can be rejected. The *Q* statistic (*Q* = 54.06, *df* = 16, *p* < 0.001) indicated that the effect sizes differed significantly across the studies. *I*^2^ of 72% suggests that most of the observed variance was due to differences on a study-level. *T*^2^ of 0.12 suggests a small amount of absolute dispersion. Calculation of the 95% PI [− 0.67, − 0.25] suggests that the true effect size of a similar future study would fall within this range in 95% of the time. Most of the PI lies in the negative range, thereby indicating that interventions would be effective in most settings [30, 31].

### Meta-analysis: effectiveness of interventions to enhance emotion regulation

**Fig. 3:**
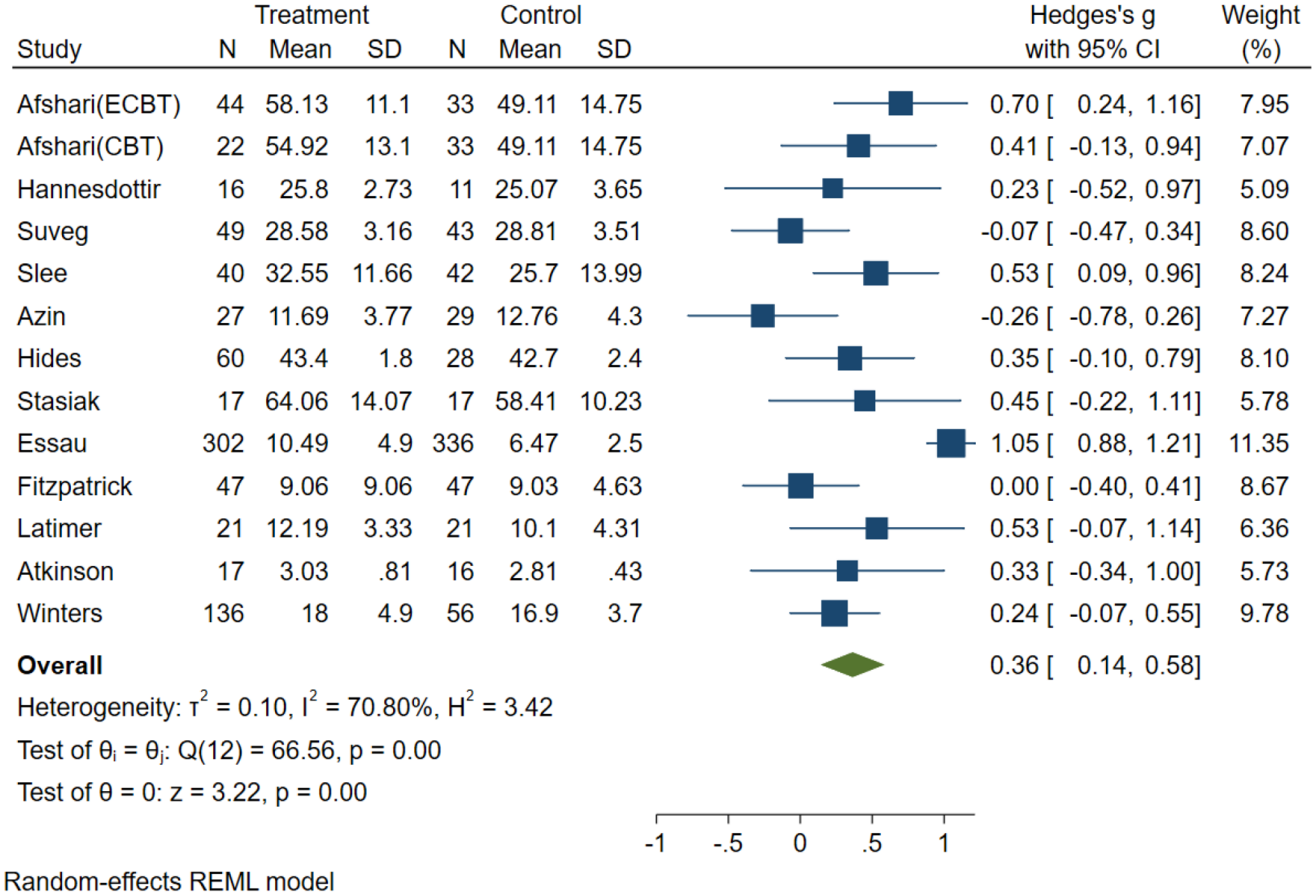
Forest plot: random-effects model (reduced) with emotion regulation as primary outcome

The original random effects model was based on 14 effect sizes from 13 independent studies with ER abilities as an outcome. The full model indicates a treatment effect of (*g* = 0.43, 95% CI [0.18, 0.69], *p* < 0.001).The metaninf and the Galbraith plot suggested two influential studies, Livheim et al. [40] and Essau et al. [41] (see supplementary material Plots 3 and 4). In comparison to Essau et al. (*N* = 638), the study by Livheim (*N* = 25) was significantly underpowered, hence we decided to remove this study from the following analysis. (Results of the full and the reduced model are both presented in Table 3). The forest plot of the reduced model indicated that three studies [41–43] showed a significant positive effect, while the remaining 10 studies had no significant effects (see Fig. 3). Overall, the results suggest a small treatment effect (*g* = 0.36, 95% CI [0.14, 0.58], *p* < 0.001). Based on the CI and the *z* statistic (*z* = 3.22, *p* < 0.001), the null hypothesis that the intervention has no impact on ER was rejected. The *Q* statistic (*Q* = 66.56, *df* = 12, *p* < 0.001) suggests that effect sizes differed significantly across the studies. *I*^2^ of 70.8% suggests that most of the observed variance was due to differences on a study level (e.g., sampling error). *T*^2^ of 0.10 suggests a small amount of between-study variance. The 95% PI = [0.14, 0.58] is in the positive range, suggesting that future studies will most likely find a positive effect size within this range [30, 31].

**Table 3:** Random effect models and sub-group analyses with emotion regulation as outcome

Reduced data set	*m*	*k*	*n*	Hedges *g*	95% CI	*p* (*z* test)	*Q*	*p* (*Q*)	*T*^2^	*I*^2^ (%)
Emotion regulation	13	12	1513	0.36	0.14, 0.58	0.00	66.56	0.00	0.010	70.80
Emotion regulation by										
Intervention										
CBT intervention	8	8	969	0.45	0.15, 0.75		35.96	0.00	0.12	71.32
ER intervention	4	4	269	0.22	− 0.15, 0.58		58.86	0.06	0.08	58.86
Control group										
Active control	8	8	521	0.20	− 0.01, 0.42		9.99	0.19	0.03	32.10
Passive control	5	4	992	0.57	0.22, 0.93		25.71	0.00	0.12	77.35
Quality rating										
Strong	1	1	82	0.53	0.09, 0.96		0.00			–
Moderate	7	7	1148	0.29	− 0.07, 0.65		63.03	0.00	0.19	84.30
Weak	5	4	283	0.44	0.20, 0.68		1.82	0.77	0.00	0.00
Full-data set										
Emotion regulation	14	13	1538	0.43	0.18, 0.69	0.00	77.82	0.00	0.16	77.89
Emotion regulation by										
Intervention										
CBT intervention	7	7	969	0.58	0.30, 0.85		59.96	0.00	0.07	59.96
ER intervention	5	5	321	0.57	− 0.17, 1.32		23.53	0.00	0.63	90.13
Control group										
Active control	9	9	546	0.37	0.01, 0.73		26.64	0.00	0.22	75.35
Passive control	5	4	992	0.57	0.22, 0.93		25.71	0.00	0.12	77.35
Quality rating										
Strong	1	1	82	0.53	0.09, 0.96		0.00			–
Moderate	7	7	1148	0.29	− 0.07, 0.65		63.03	0.00	0.19	84.30
Weak	5	4	308	0.63	0.16, 1.10		14.38	0.01	0.24	72.89

### Heterogeneity and bias assessment

To explore possible causes of heterogeneity and investigate whether effect sizes varied for certain subgroups, a meta-regression and subgroup analyses were conducted (see Tables 2, 3, 4).

**Table 4:** Meta- regression with effect size as dependent variable and potential moderators as predictors

Predictor variables	Emotion dysregulation					Emotion regulation				
	*β*	SE	*z*	*p*	95% CI	*β*	SE	*z*	*p*	95% CI
Intercept	− 0.48	0.19	− 2.54	0.01	− 0.86,− 0.11	1.24	0.56	2.19	0.02	0.13, 2.35
Intervention										
CBT intervention										
ER intervention	− 0.27	0.21	− 1.32	0.18	− 0.69, 0.13	0.23	0.29	0.81	0.41	− 0.33, 0.81
Control group										
Active control										
Passive control	0.24	0.21	1.18	0.23	− 0.16, 0.66	− 0.63	0.32	− 1.92	0.05	− 1.27, 0.01
Quality rating										
Strong										
Moderate	0.07	0.36	0.22	0.82	− 0.63, 0.79	− 0.42	0.35	− 1.20	0.23	− 1.11, 0.26
Weak	− 0.72	0.52	− 1.39	0.16	− 1.75, 0.29	− 0.34	0.34	− 1.01	0.31	− 1.01, 0.32
Age group										
Child	0.55	0.46	1.20	0.23	− 0.35, 1.45	− 0.54	0.41	− 1.32	0.18	− 1.35, 0.26
Early adolescence	0.23	0.42	0.55	0.58	− 0.60, 1.07	0.20	0.36	0.56	0.57	− 0.51, 0.92
Adolescence										
Late adolescence	0.13	0.41	0.32	0.75	− 0.68, 0.95	− 0.08	0.29	− 0.29	0.77	− 0.66, 0.49

### Moderator meta-regression

#### Emotion dysregulation

The meta-regression model with effect size (*k* = 17) as the dependent variable and age group, intervention type, quality of study and control group as predictor variables, was non-significant (*χ*^2^ = 14.37, *p* = 0.07) thereby suggesting that none of the coefficients in the model, apart from the intercept, are significantly different from zero. Similarly, none of the moderators had a significant impact on the overall effect size. Furthermore, the *I*^2^ index (66%) suggest a moderate level of heterogeneity in the model and that only 31.5% of the between-study variance is explained by the moderators (*R*^2^ = 31.47). Based on the meta-regression results none of the included study-level factors seem to influence the overall effect-size. However, with respect to recent meta-regression recommendations, one should not conclude that a covariate is unrelated to the effect size if there are less than ten studies per covariate [44]. Consequently, we explore this further relationship further in the subgroup analyses.

#### Emotion regulation

The meta-regression model with effect size (*k* = 13) as the dependent variable was significant (*χ*^2^ = 20.58, *p* < 0.05) thereby suggesting that at least one of the coefficients in the model, apart from the intercept, is significantly different from zero. The results indicate that the control group variable had a significant impact on effect size (see Table 4). The *I*^2^ index (40%) suggest a moderate to small level of heterogeneity in the model and that 75% of the between-study variance is explained by the moderators in the model (*R*^2^ = 75.09). As stated above, due to the limited amount of studies per covariate in the model, the following subgroup-analyses were conducted to explore this relationship further.

### Subgroup analysis: type of intervention

#### Emotion dysregulation

The results indicate that for individuals who received a specific ER intervention, emotion dysregulation decreased by *g* = − 0.51, and in non-specific interventions emotion dysregulation decreased by *g* = − 0.40. This suggests that interventions with a greater focus on ER could be more effective in reducing ER difficulties. However, the large amount of heterogeneity (*I*^2^ = 70% and 59%) makes direct comparisons between the subgroups difficult. This is also supported by the non-significant test of group differences (*Q*_b_ (2) = 0.36, *p* = 0.84) (see Fig. 6 in supplements).

#### Emotion regulation

The results indicate that for individuals who received a specific ER intervention, emotion regulation improved by *g* = 0.22, and in non-specific interventions emotion regulation improved by *g* = 0.45. Heterogeneity is large for all subgroups (71% and 58%) and the test of group difference non-significant (*Q*_b_ (2) = 1.29, *p* = 0.51). Furthermore, one of the subgroups only consisted of fours studies, which has been considered as too small to derive definite conclusions (see Fig. 7 in supplements).

### Subgroup analysis: type of control group

#### Emotion dysregulation

The results indicate that for studies with an active control condition ED decreased by *g* = − 0.19, while for studies with passive control conditions ED decreased by *g* = − 0.66. The significant *Q* statistic (*Q* = 6.88, *df* = 1, *p* < 0.001), suggests that the true mean effect varies depending on the type of control condition. Heterogeneity within the active control subgroup was significantly lower (*I*^2^ = 39%) compared to the passive control subgroup (*I*^2^ = 71%). Thus differentiating between types of control groups partially explained the level of heterogeneity (see Fig. 8 in supplements).

#### Emotion regulation

Similarly, for ER effect sizes, studies with an active control condition improved ER by *g* = 0.20, while for studies with passive control conditions ER improved by *g* = 0.57. The significant *Q* statistic (*Q* = 3.09, *df* = 1, *p* < 0.001), suggests that the true mean effect varies depending on the type of control condition. Heterogeneity within the active control subgroup was significantly lower (*I*^2^ = 32%) compared to the passive control subgroup (*I*^2^ = 77%, see Fig. 9 in supplements)).

### Subgroup analysis: type of disorder and ER strategy

Subgroup analyses for different types of disorders and different ER strategies were conducted, but due to insufficient numbers of studies (*n* ≤ 4) in the respective subgroups no meaningful interpretations were possible. (Results of these are provided in the supplementary materials, see Figs. 10 and 11.)

### Subgroup analysis: age group

#### Emotion dysregulation

Subgroup analyses for different age groups indicate that that ED decreased by *g* = − 0.16 in children, *g* = − 0.62 in early adolescence, *g* = − 0.45 in adolescents and *g* = − 0.59 in late adolescents. Heterogeneity is large for all subgroups (50–89%) and the test of group difference non-significant (Q_b_ (3) = 1.28, *p* = 0.73). Furthermore, apart from the age group “adolescence” all other subgroups only consisted of 2–3 studies, which has been considered as too small to derive definite conclusions (see Fig. 12 in supplementary materials).

#### Emotion regulation

Subgroups in this analysis did not exceed more than four studies per group, which is suggested to be too small in order to derive meaningful interpretations. (Results of these are provided in the supplementary materials, see Fig. 13.)

### Subgroup analyses: quality rating

#### Emotion dysregulation

Studies (*k* = 7) with strong quality ratings decreased ED by *g* = − 0.59, which was higher than the overall effect-size *g* = − 0.46. Studies of moderate quality (*k* = 6) had smaller effect sizes *g* = − 0.13, while studies with the lowest quality ratings (*k* = 2) decreased ED by *g* = − 0.81.

#### Emotion regulation

For ER only one study was rated as strong (*g* = 0.53), while the other studies were moderate (*k* = 7, *g* = 0.29) or weak (*k* = 4, *g* = 0.44). Due to the limited number of studies, we recommend that these results are treated with caution.

### Publication bias

**Fig. 4:**
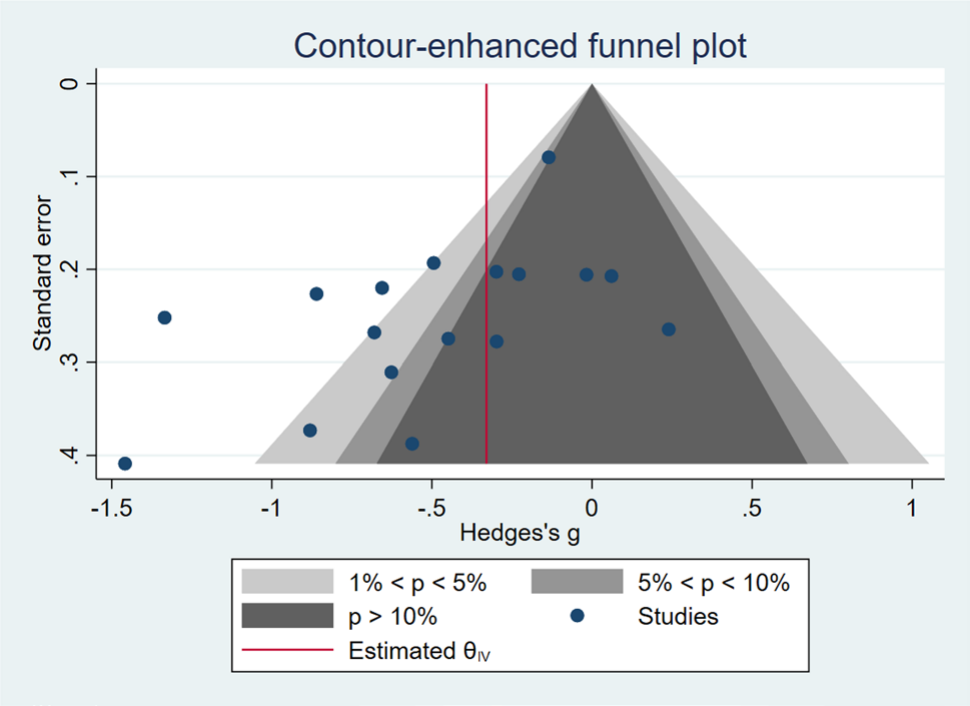
Funnel plot to detect publication bias

The contour-enhanced funnel plot (see Fig. 4) shows an asymmetric pattern. Visual inspection of the funnel plot indicates more studies on the left side. Furthermore, we see missing data points at the top and bottom of the funnel, for both the significant (light grey) and non-significant (dark grey) areas. In the case of a publication bias, we would expect to see missing studies in the non-significant areas. The present funnel plot seems to rather suggest a gap for studies including larger sample sizes. Most of the studies included in this review involved similar, small to medium-size samples (great density in the middle), which can result in spuriously increased effect sizes. Therefore, we conducted the Egger’s test, which was significant, thereby suggesting a bias, due to small-study effects (*z* = − 2.22, *p* < 0.05).It has been reported however that funnel-plot asymmetry can be caused by publication bias, as well as other factors such as poor methodological quality or between study heterogeneity [32]. Due to the large amount of heterogeneity in our analysis we performed the Egger’s test again, this time taking into account between-study heterogeneity, as a result of different types of interventions, ER measures and control groups. We found that heterogenity due to different intervention types, significantly influenced the results of the the Egger’s test, which was nonsignificant when intervention type was added to the model (*z* = − 1.31, *p* = 0.19).

### Sensitivity analyses

#### Effect of heterogeneity

Due to the large amount of heterogeneity in the presented models, we conducted further sensitivity analyses to test the robustness of our results. Hence, we fixed the value *I*^2^ to 10% to represent a small level of heterogeneity. The result suggest that with a smaller level of heterogeneity there is a smaller, but significant effect size of *g* = − 0.33 (*z* = − 6.64, *p* < 0.001) with a 95% CI of [− 0.46, − 0.23] for emotion dysregulation. The same analysis was performed for the emotion regulation model, indicating that lower heterogeneity would result in a larger effect size of *g* = 0.57 (*z* = 8.3*,* *p* < 0.001) with a 95% CI [0.47, 0.68]. These results suggest that heterogeneity has an impact on the overall effect size, but also that current interventions effectively improve emotion regulation processes whether heterogeneity is small or large.

### Meta-regression: are changes in ER associated with changes in psychopathology?

**Fig. 5:**
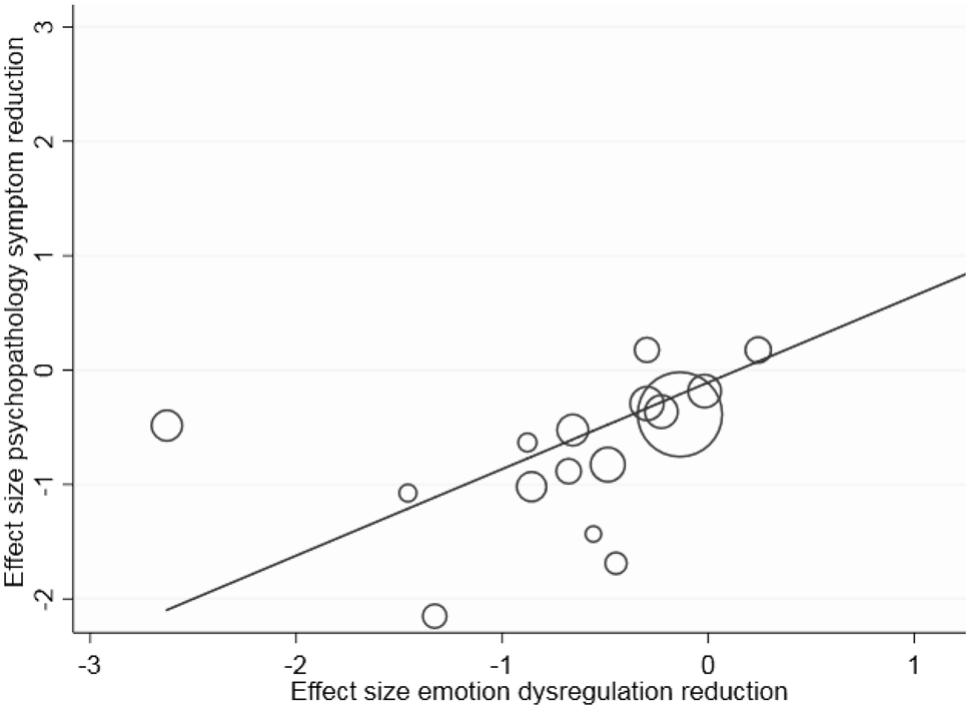
Meta-regression: showing significant positive relationship between reduced emotion regulation problems and reduced psychopathology

**Fig. 6:**
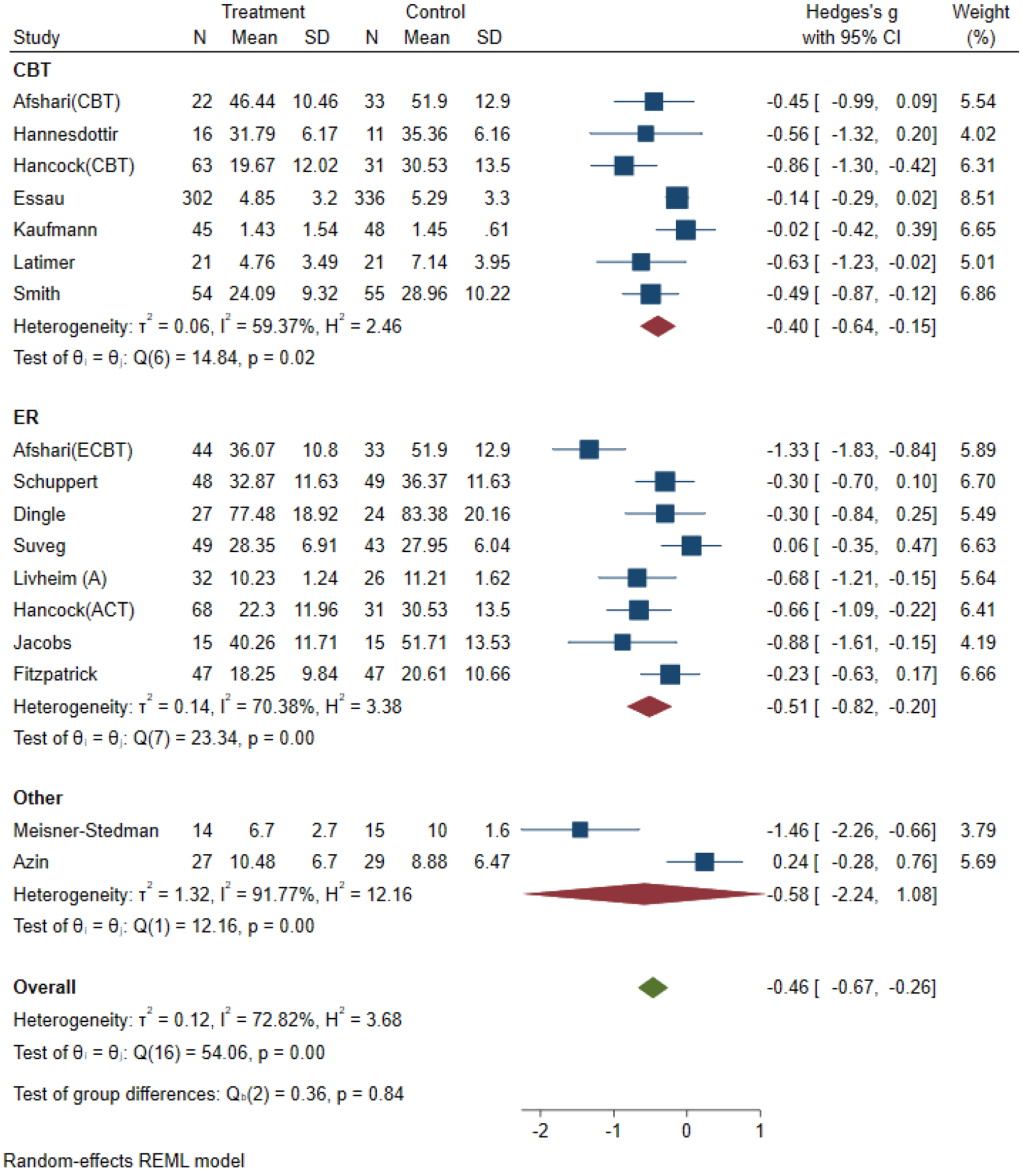
Subgroup analysis of type of intervention for emotion dysregulation

**Fig. 7:**
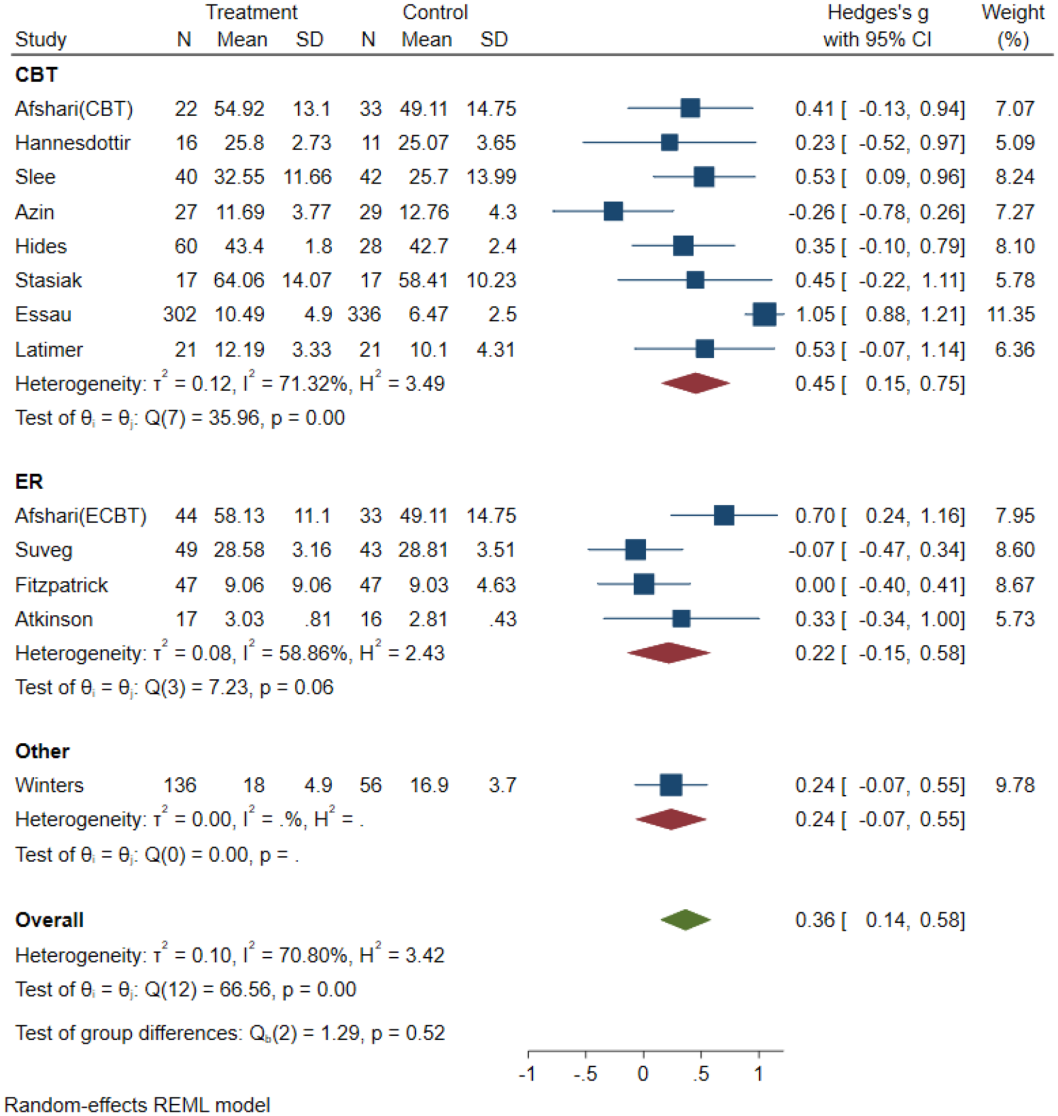
Subgroup analysis of type of intervention for emotion regulation

**Fig. 8:**
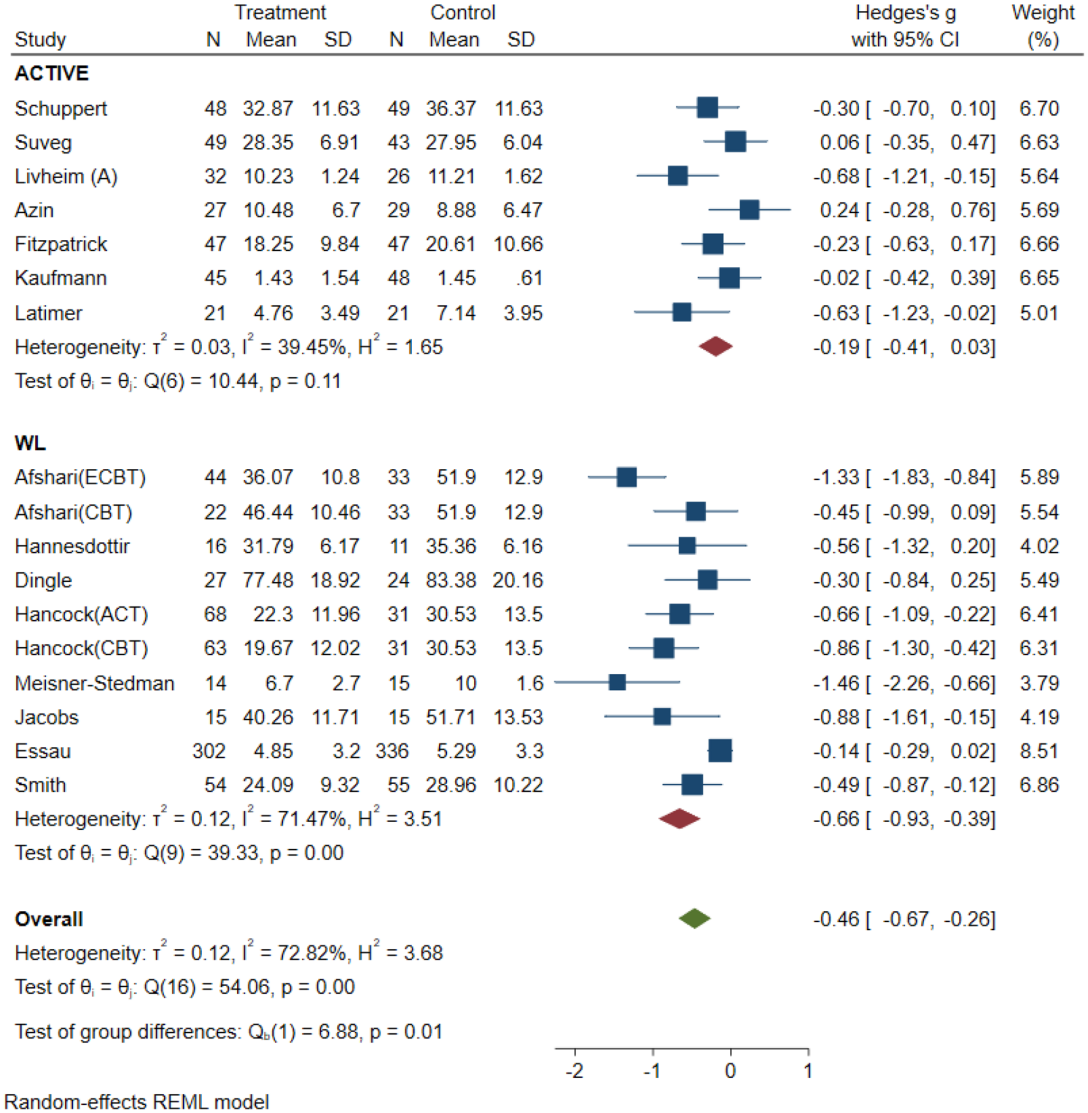
Subgroup analysis of type of control group for emotion dysregulation

**Fig. 9:**
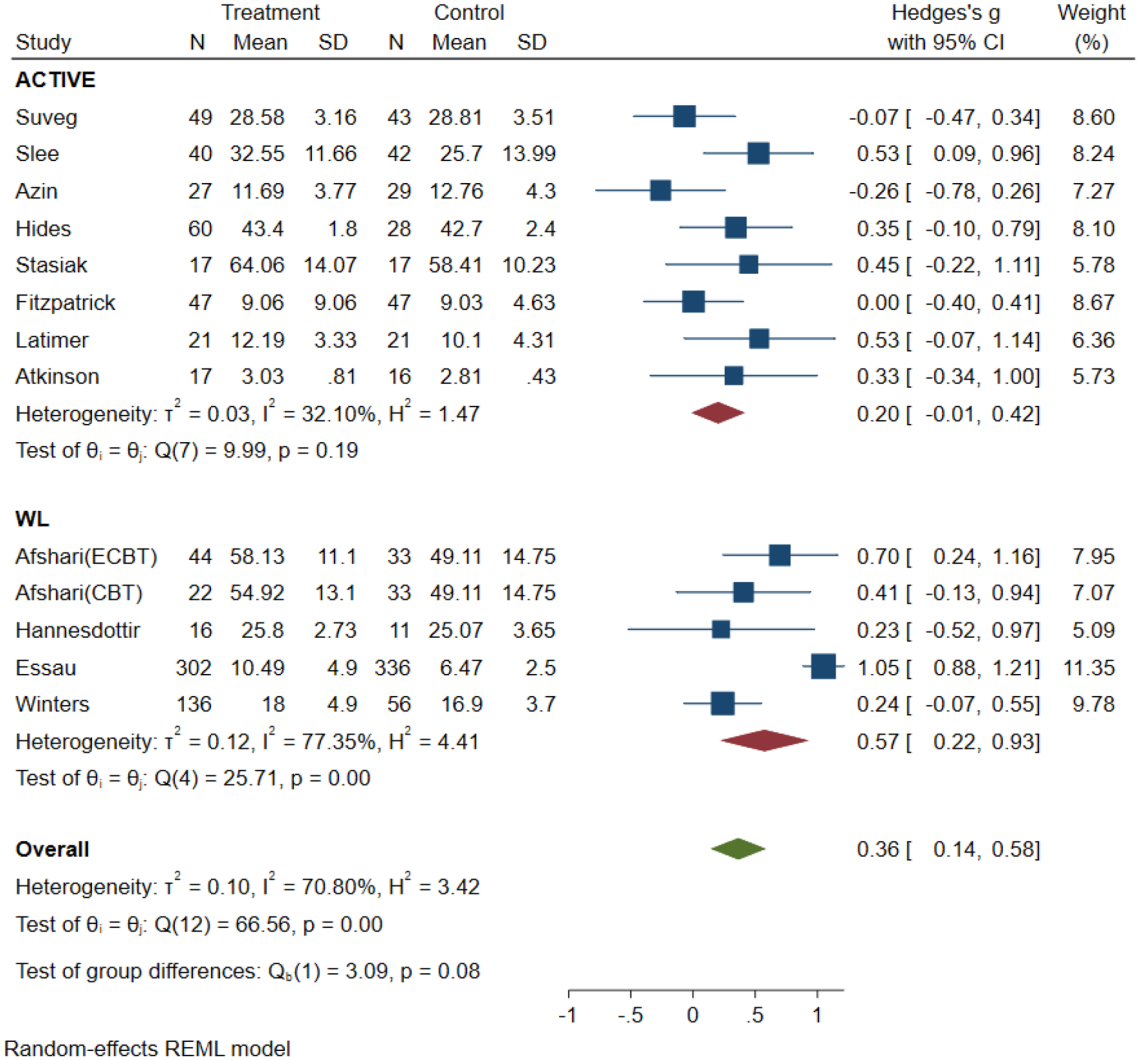
Subgroup analysis of type of control group for emotion regulation

Only two studies reported whether changes in ER were associated with changes in psychopathology. Slee et al. [43] investigated adolescents engaging in deliberate self-harm, and found that changes in ER difficulties partially mediated decreases in deliberate self-harm. The second study [45] found that changes in acceptance mediated decreases in anxiety and depression. Our meta-regression indicated a significant positive relationship between larger effect sizes of reduced ED and larger effect sizes of reduced psychopathology (see Fig. 5; *β* = 0.76, *t* = 2.93, *p* = 0.01). In other words, studies showing greater effectiveness in reducing ER difficulties were also more effective in reducing psychopathological symptoms (Figs. 6, 7, 8, 9).

## Discussion

The results of the meta-analyses suggested small to medium effect sizes for current interventions to improve ER in youth, regardless whether the full or reduced data set was employed. For emotion dysregulation effect sizes ranged between *g* = − 0.46 and *g* = − 0.52, and for emotion regulation effect sizes ranged between *g* = 0.36 and *g* = 0.43. Furthermore, our findings indicated that interventions, which effectively improved psychopathology also improved ER difficulties. These results are in line with the adult literature [19] showing that interventions which effectively improved ER difficulties also decreased psychopathology. Our results indicated that the type of control group had a significant impact on the effect size, whereby studies with a waitlist (passive) control group showed larger effect sizes in comparison to studies including an active control group. Unfortunately, the nature of the active control conditions was not always described in detail, therefore making any further conclusions difficult. The present meta-analysis adds to existing findings by synthesizing data from randomized control studies that involve children and adolescents as a target population, which has been neglected so far.

### Clinical implications

Despite the limited evidence for the causal role of ER in the development and treatment of psychopathology in youth, the present findings encourage further development and evaluation of interventions that target ER specifically.

The average effect sizes suggest that interventions effectively change ER, irrespective of the type of the intervention program. However, the validation of existing interventions represents an important area for future work. As the present systematic review demonstrates there is a significant variety across intervention protocols in the way they target emotion regulation, and it is not clear yet which of the included components effectively enhance emotion regulation. Furthermore, there is still limited evidence with respect to different age groups and psychopathologies, which is of particular importance. First, research has shown that emotion regulation does not develop in a linear pattern, but that different developmental stages are characterised by certain advancements and deficits [46, 47]. For instance Cracco et al. [46] and Zimmermann et al. [47] have demonstrated that there is a significant shift in adolescents’ emotion regulation patterns (e.g., access to strategies, use of adaptive vs maladaptive strategies), which current interventions do not seem to take into consideration. Therefore, we argue that more efforts need to be made to increase our understanding of what works for who and when, so that relevant changes can be implemented in current clinical treatment plans. Secondly, young people frequently display a wide range of psychopathological symptoms and comorbidities [48, 49]. This can make interventions that have been designed for single-disorder symptoms, less suitable for this group. Thus the present review supports existing recommendations that ER interventions are effective in reducing a wider range of psychopathological symptoms by targeting underlying processes, which makes them highly suitable for young populations with high rates of comorbidities [17]. Furthermore, our results suggests the potential of transdiagnostic treatments being added as adjunctive modules in existing treatment protocols. This approach has already found support in adult studies where ER interventions in combination with CBT have resulted in better mental health and wellbeing outcomes than CBT alone [50].

### Strengths and limitations

The results were based on a relatively small number of studies, which primarily involved small to medium sized samples. It can be assumed that the variety in populations, intervention settings (e.g., digital, inpatient and outpatient, schools) and use of ER measures lead to large between-study variation, which may have biased our findings. With respect to the latter it has been highlighted recently that meta-analyses with an increased psychometric focus could provide more insights regarding the impact of measurement error on outcome biases [51]. In the present meta-analysis, only 11 of the 19 studies reported information on reliability, which did not allow us to correct for measurement error. Hence, we highly encourage future meta-analysts to also consider bias due to measurement error. Moreover, there was a great variety between interventions, even though CBT formed the basis of most interventions. However, due to the limited amount of data available, it was impossible to provide further insights regarding the impact of these study artifacts on the overall effect size.

Furthermore, due to missing evidence from longitudinal mediation analyses, the present study could only partly address the second research question whether changes in ER precede changes in psychopathology. Only two studies [43, 45] reported whether changes in ER were associated with changes in psychopathology. Both studies found that changes in ER mediated decreases in psychopathology. Similarly, our meta-regression showed a significant positive relationship between effect sizes of improved ER difficulties and effect sizes of improved psychopathology. Moreover, most studies only assessed changes in anxiety or depression even though a wider range of symptoms was reported at baseline. Due to the current lack of research reporting on ER outcomes in relation to different psychopathology outcomes, we were not able to conduct more specific mediation analyses. Similar issues have been raised in previous systematic reviews [52]. We recommend that future research includes measures of ER so that underlying mechanisms of change can be identified.

The quality of the included studies ranged from weak to strong. Even though we focused primarily on RCTs, there was a significant lack of high quality studies. The limited evidence may have made it difficult to detect differences in effect sizes relating to study quality. Moreover, it has frequently been pointed out that the level of quality found in primary research has a significant impact on the quality of any systematic review, due to the fact that systematic reviews rely on data from existing studies. Following this we can only emphasize that future research needs to focus on the delivery of more high quality studies that provide high-quality research outcomes. In line with this, we acknowledge that while we had hoped to identify more high-quality studies by excluding non-peer-reviewed articles, the exclusion of such unpublished data may have resulted in biased outcomes. Although our publication bias assessment did not clearly indicate the presence of a publication bias, this may have been due to the high level of heterogeneity. However, we would like to highlight that there was significant lack of large-sample size studies that included a comprehensive psychopathology assessment and targeted youth populations.

### Future suggestions

Further RCTs including larger sample sizes, different age groups and mental disorders are needed. While evidence suggests that research has widely neglected populations under the age of 25, future research should specifically address youth populations between the ages of 10 and 12 years. They form an interesting age group as research has emphasized a significant drop in ER skills at this age [47]. Furthermore, studies involving youth mostly investigate anxiety or depressive symptoms, while only a few have looked at ER in relation to other mental disorders. Similarly, interventions with a specific focus on ER often target specific disorders. Considering the suggested transdiagnostic nature of ER, future studies should involve participants from a broader psychopathological spectrum.

To increase our understanding of ER interventions and associated change mechanisms, future research needs to assess and actually report ER processes. A large number of studies was excluded, due to missing ER assessment. This can not only improve future interventions, but would also reduce the exploratory nature of current interventions. In line with this we suggest that future research should focus on the impact of measurement error in their studies. As mentioned above, studies included a wide range of ER measures, which have been based on different theories and models around ER. Thus, a psychometric meta-analysis of current ER measures, would be highly beneficial to the field.

Finally, we found that the investigation of positive ER strategies and ER abilities is still widely neglected. Although, past research has highlighted that adaptive ER strategies, as opposed to maladaptive strategies, were more strongly related to psychopathology in youth [53]. The opposite has been reported in adult studies [54]. We identified only one study that assessed a positive ER strategy [55]. This could be related to the fact that positive psychology is still a rather young field in comparison to the traditional CBT approaches or that the use of ER strategies has been less frequently studied in youth populations. Nevertheless, in line with previous research [12, 53] and our findings, we argue for a greater focus on the positive dimension of ER especially in researching and working with young populations.

## Conclusion

This is the first meta-analysis that summarizes the evidence of psychological intervention to enhance ER in youth. The findings indicate that current interventions improve ER and that changes in ER co-occur with changes in psychopathological symptoms. The findings add to the existing literature, which has widely neglected youth populations thus far. Important implications for future clinical work and research have been made.

## Electronic supplementary material

Below is the link to the electronic supplementary material.

Supplementary file1 (DOCX 904 kb)
