# Supplementary material for: Effectiveness of current psychological interventions to improve emotion regulation in youth: a meta-analysis
Source: Eur Child Adolesc Psychiatry. 2020 Feb 27;30(6):829–48. doi: 10.1007/s00787-020-01498-4 (PMC8140974; doi:10.1007/s00787-020-01498-4)
Supplement: Supplementary file 1 — Supplementary file1 (DOCX 904 kb) [file 787_2020_1498_MOESM1_ESM.docx]

# Supplementary materials

## References of studies included in the meta-analysis

Afshari, A., Neshat-Doost, H. T., Maracy, M. R., Ahmady, M. K., & Amiri, S. (2014). The effective comparison between emotion-focused cognitive behavioral group therapy and cognitive behavioral group therapy in children with separation anxiety disorder. *Journal of research in medical sciences*, *19*(3), 221–227.

Atkinson, M. J., & Wade, T. D. (2016). Does mindfulness have potential in eating disorders prevention? A preliminary controlled trial with young adult women. *Early intervention in psychiatry*, *10*(3), 234–245. https://doi.org/10.1111/eip.12160

Azrin, N. H., Donohue, B., Teichner, G. A., Crum, T., Howell, J., & DeCato, L. A. (2001). A Controlled Evaluation and Description of Individual-Cognitive Problem Solving and Family-Behavior Therapies in Dually-Diagnosed Conduct-Disordered and Substance-Dependent Youth. *Journal of Child and Adolescent Substance Abuse*, *11*(1), 1–43. https://doi.org/10.1300/J029v11n01

Dingle, G. A., & Fay, C. (2017). Tuned In: The effectiveness for young adults of a group emotion regulation program using music listening. *Psychology of Music*, *45*(4), 513–529. https://doi.org/10.1177/0305735616668586

Fitzpatrick, K. K., Witte, T. K., & Schmidt, N. B. (2005). Randomized controlled trial of a brief problem-orientation intervention for suicidal ideation. *Behavior therapy*, *36*(4), 323–333. https://doi.org/10.1016/S0005-7894(05)80114-5

Hancock, K. M., Swain, J., Hainsworth, C. J., Dixon, A. L., Koo, S., & Munro, K. (2016). Acceptance and Commitment Therapy versus Cognitive Behavior Therapy for Children With Anxiety: Outcomes of a Randomized Controlled Trial. *Journal of Clinical Child & Adolescent Psychology*, *4416*(March), 1–16. https://doi.org/10.1080/15374416.2015.1110822

Hannesdottir, D. K., Ingvarsdottir, E., & Bjornsson, A. (2017). The OutSMARTers Program for Children With ADHD: A Pilot Study on the Effects of Social Skills, Self-Regulation, and Executive Function Training. *Journal of attention disorders*, *21*(4), 353–364. https://doi.org/10.1177/1087054713520617

Hides, L. M., Elkins, K., Scaffidi, A., Cotton, S. M., Carroll, S., & Lubman, D. I. (2011). Does the addition of integrated cognitive behaviour therapy and motivational interviewing improve the outcomes of standard care for young people with comorbid depression and substance misuse? *The Medical Journal of Australia*, *195*(June).

Jacobs, R. H., Watkins, E. R., Peters, A. T., Feldhaus, C. G., Barba, A., Carbray, J., & Langenecker, S. A. (2016). Targeting Ruminative Thinking in Adolescents at Risk for Depressive Relapse: Rumination-Focused Cognitive Behavior Therapy in a Pilot Randomized Controlled Trial with Resting State fMRI. *PloS One*, *11*(11), e0163952. https://doi.org/https://dx.doi.org/10.1371/journal.pone.0163952

Kaufman, N. K., Rohde, P., Seeley, J. R., Clarke, G. N., & Stice, E. (2005). Potential mediators of cognitive-behavioral therapy for adolescents with comorbid major depression and conduct disorder. *Journal of consulting and clinical psychology*, *73*(1), 38–46. https://doi.org/10.1037/0022-006X.73.1.38

Latimer, W. W., Winters, K. C., D’Zurilla, T., & Nichols, M. (2003). Integrated Family and Cognitive-Behavioral Therapy for adolescent substance abusers: A Stage I efficacy study. *Drug and Alcohol Dependence*, *71*(3), 303–317. https://doi.org/10.1016/S0376-8716(03)00171-6

Livheim, F., Hayes, L., Ghaderi, A., Magnusdottir, T., Hogfeldt, A., Rowse, J., … Tengstrom, A. (2015). The Effectiveness of Acceptance and Commitment Therapy for Adolescent Mental Health: Swedish and Australian Pilot Outcomes. *JOURNAL OF CHILD AND FAMILY STUDIES*, *24*(4), 1016–1030. https://doi.org/10.1007/s10826-014-9912-9

Marieke Schuppert, H., Timmerman, M. E., Bloo, J., Van Gemert, T. G., Wiersema, H. M., Minderaa, R. B., … Nauta, M. H. (2012). Emotion regulation training for adolescents with borderline personality disorder traits: A randomized controlled trial. *Journal of the American Academy of Child and Adolescent Psychiatry*, *51*(12), 1314–1323.e2. https://doi.org/10.1016/j.jaac.2012.09.002

Meiser-Stedman, R., Smith, P., McKinnon, A., Dixon, C., Trickey, D., Ehlers, A., … Dalgleish, T. (2017). Cognitive therapy as an early treatment for post-traumatic stress disorder in children and adolescents: a randomized controlled trial addressing preliminary efficacy and mechanisms of action. *Journal of child psychology and psychiatry*, *58*(5), 623–633. https://doi.org/10.1111/jcpp.12673

Slee, N., Spinhoven, P., Garnefski, N., & Arensman, E. (2008). Emotion regulation as mediator of treatment outcome in therapy for deliberate self-harm. *Clinical Psychology & Psychotherapy*, *15*(4), 205–216. https://doi.org/10.1002/cpp.577

Smith, P., Scott, R., Eshkevari, E., Jatta, F., Leigh, E., Harris, V., … Yule, W. (2015). Computerised CBT for depressed adolescents: Randomised controlled trial. *Behaviour Research and Therapy*, *73*, 104–110. https://doi.org/10.1016/j.brat.2015.07.009

Stasiak, K., Hatcher, S., Frampton, C., & Merry, S. N. (2014). A pilot double blind randomized placebo controlled trial of a prototype computer-based cognitive behavioural therapy program for adolescents with symptoms of depression. *Behavioural and Cognitive Psychotherapy*, *42*(4), 385–401. https://doi.org/http://dx.doi.org/10.1017/S1352465812001087

Suveg, C., Jones, A., Davis, M., Jacob, M. L., Morelen, D., Thomassin, K., & Whitehead, M. (2017). Emotion-focused cognitive-behavioral therapy for youth with anxiety disorders: A randomized trial. *Journal of Abnormal Child Psychology*, No-Specified. https://doi.org/http://dx.doi.org/10.1007/s10802-017-0319-0

Winters, K. C., Fahnhorst, T., Botzet, A., Lee, S., & Lalone, B. (2012). Brief intervention for drug-abusing adolescents in a school setting: Outcomes and mediating factors. *Journal of substance abuse treatment*, *42*(3), 279–288. https://doi.org/10.1016/j.jsat.2011.08.005

## Outlier analysis


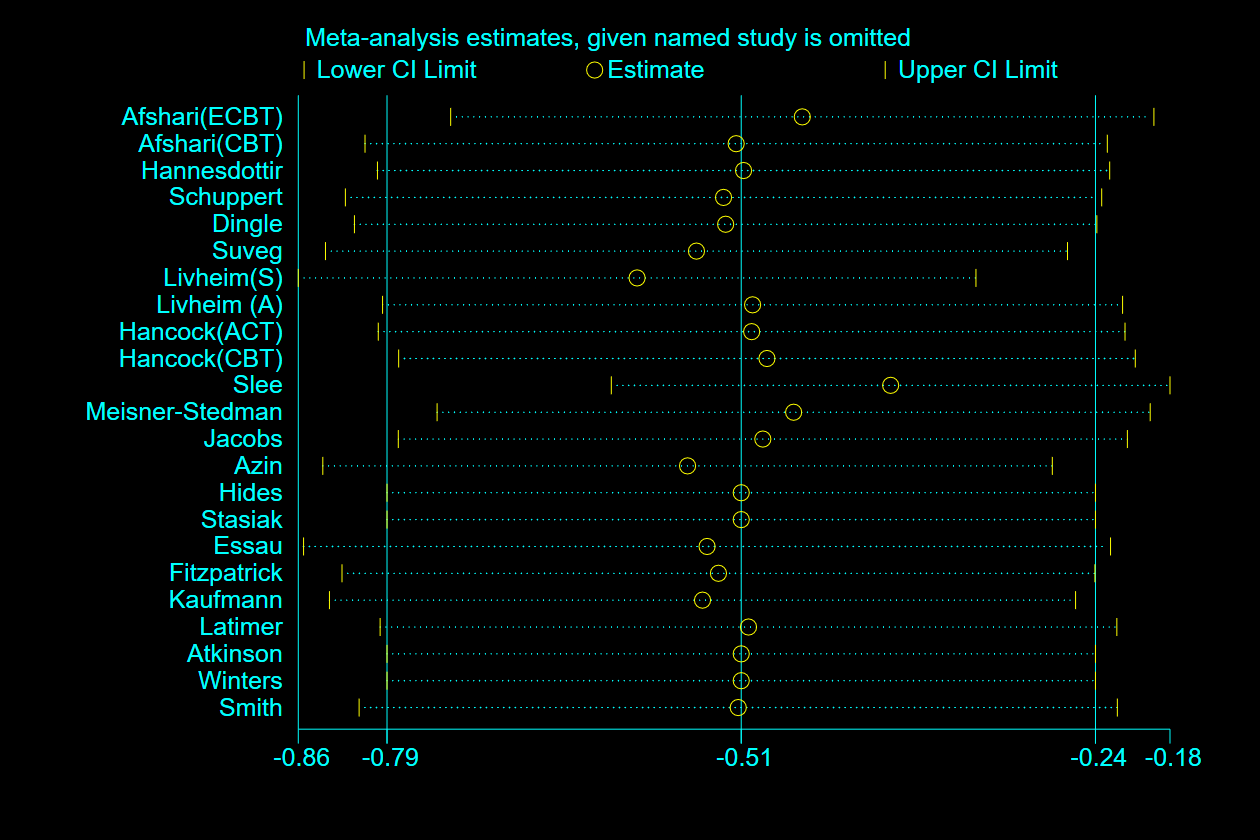


Plot 1. "Metainf" plot indicating impact of individual studies on effect size


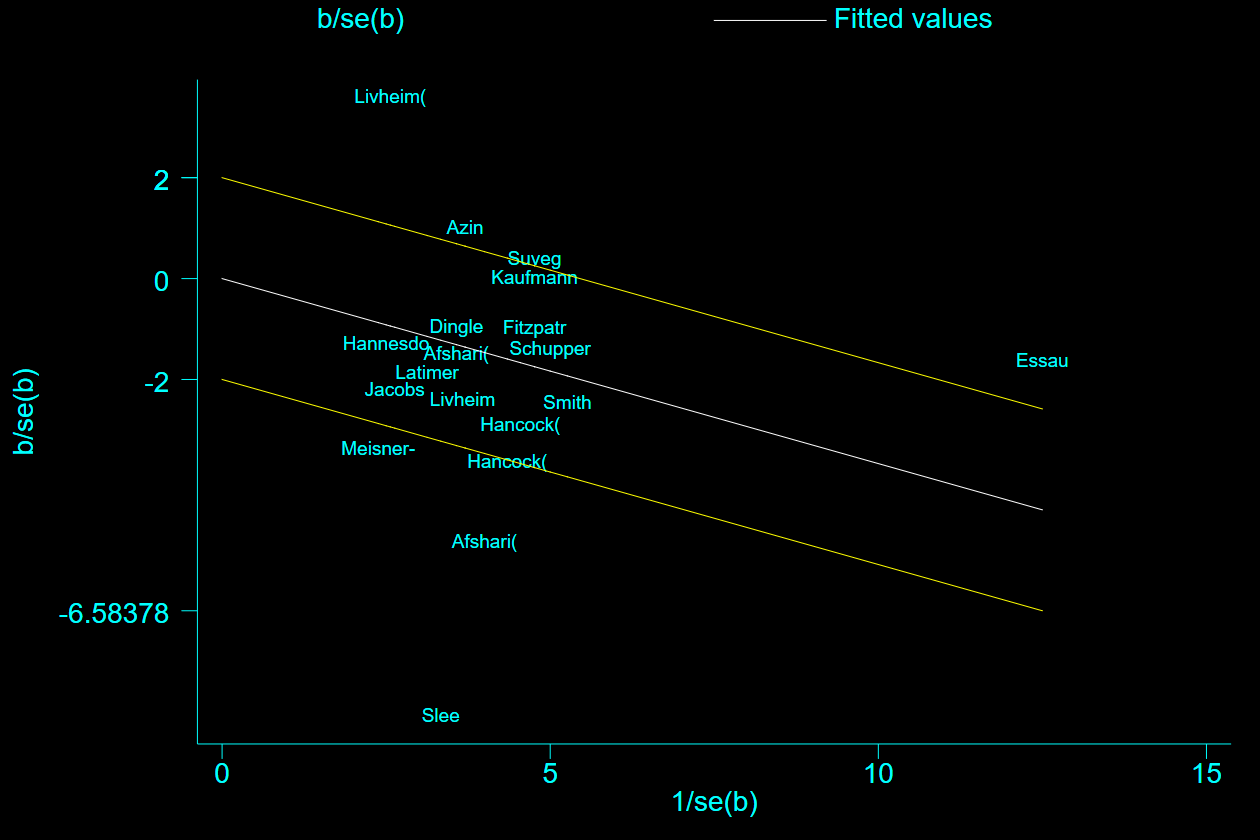


Plot 2 Galbraith plot for studies with emotion dysregulation as primary outcome


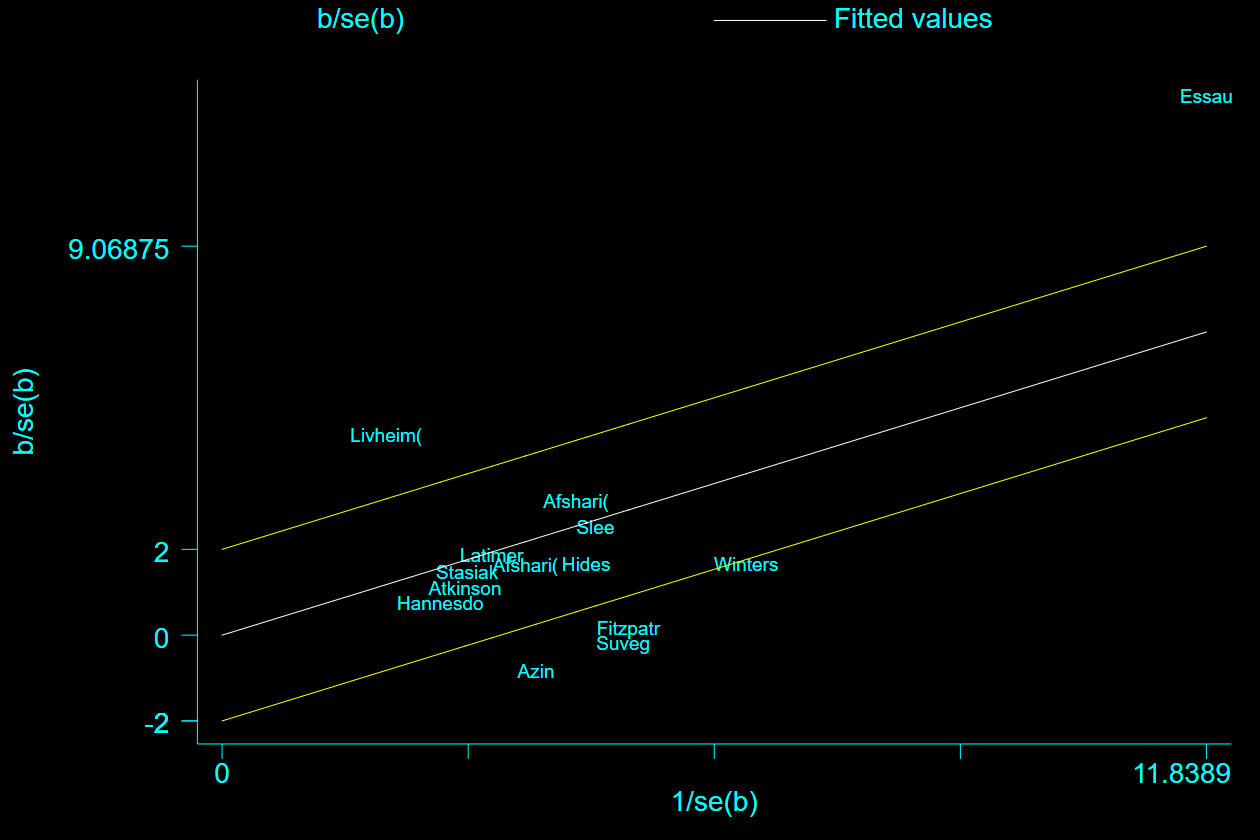


Plot 3 - Galbraith plot for studies with emotion regulation as primary outcome


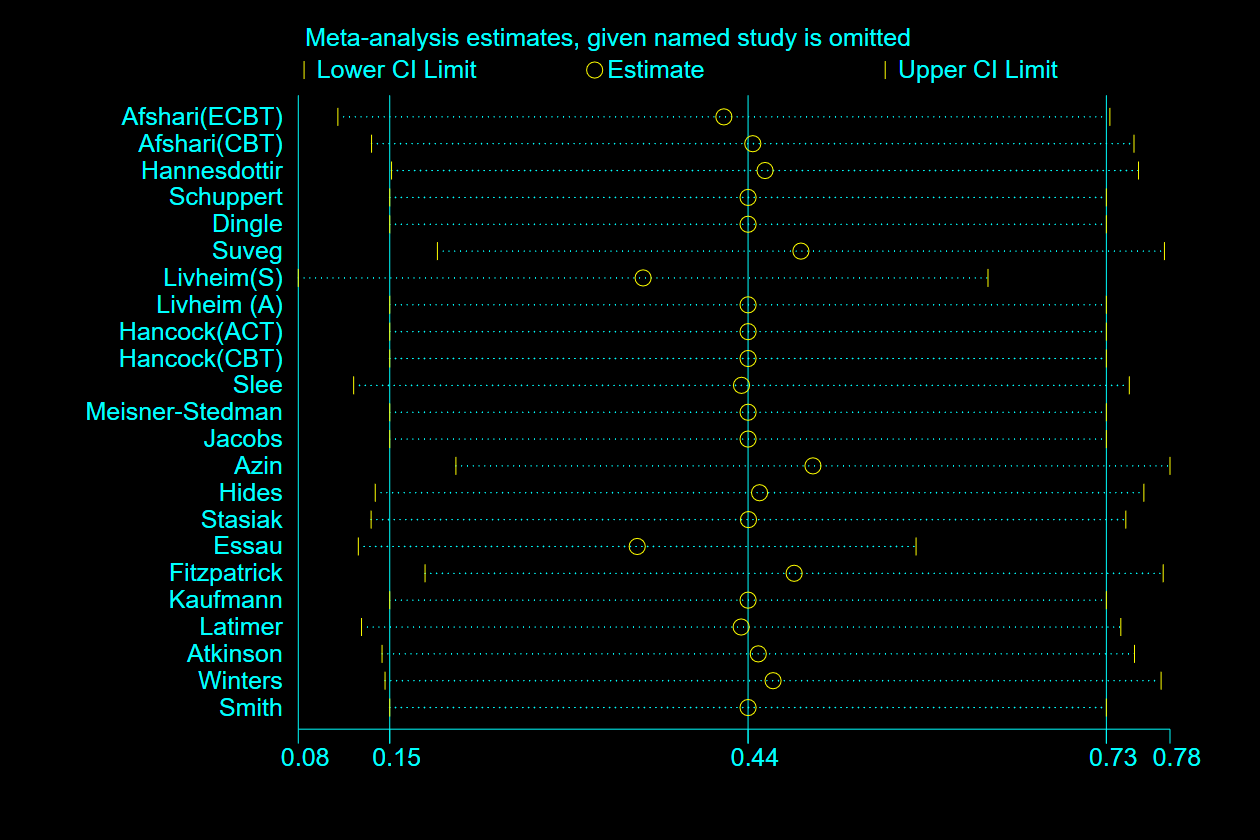


Figure 4. "Metainf" plot indicating impact of individual studies on effect size

## Further subgroup analyses


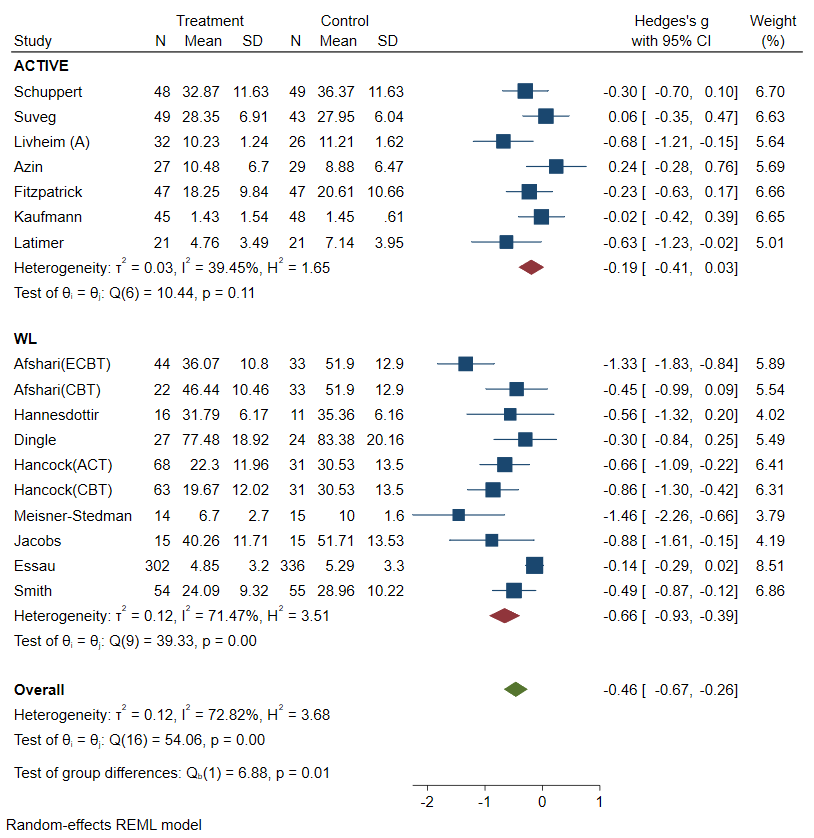


Figure 8 Subgroup analysis of type of control group for emotion dysregulation


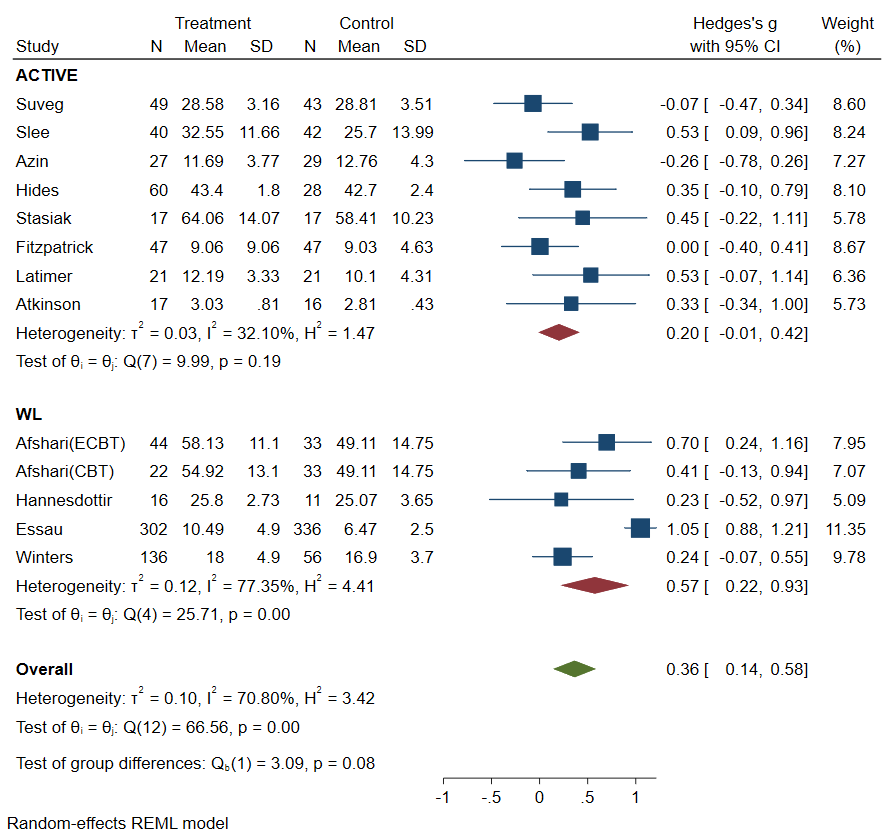


Figure 9. Subgroup analysis of type of control group for emotion regulation


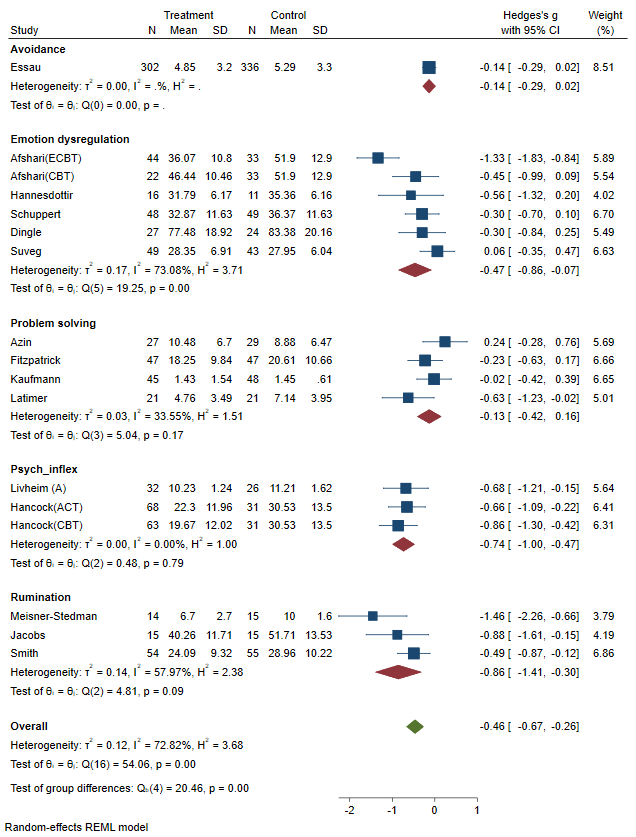


Figure 30. Subgroup analysis of type of emotion dysregulation measure


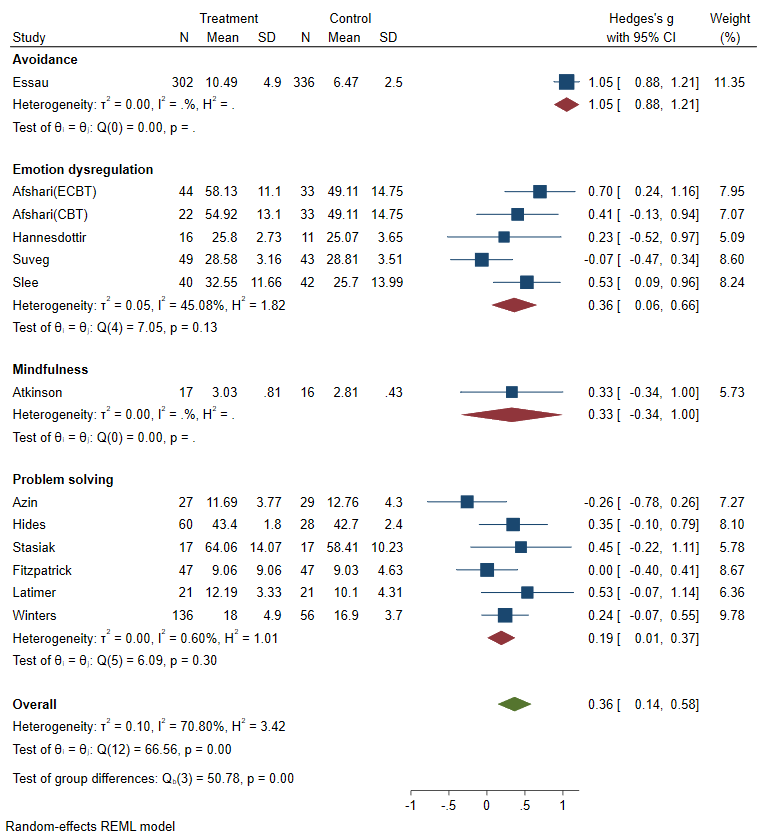


Figure 11. Subgroup analysis of emotion regulation measures


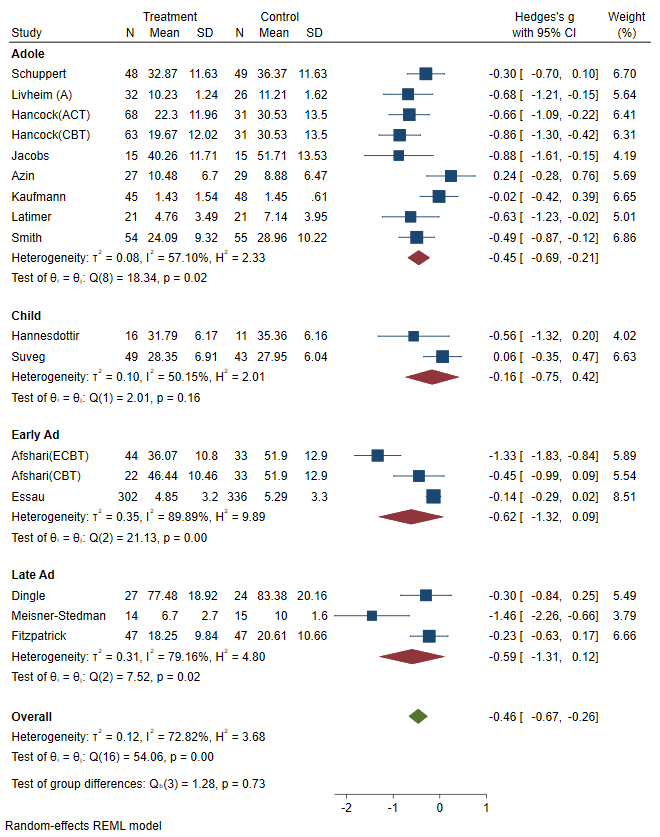


Figure 14. Subgroup analysis of age group for emotion dysregulation


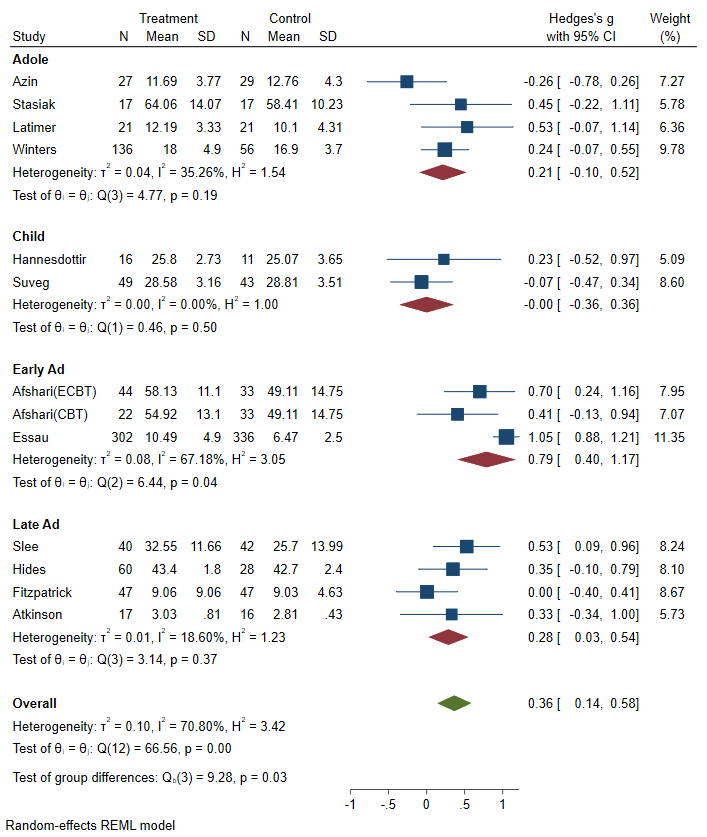


Figure 15. Subgroup analysis of age group for emotion dysregulation

Table 5. Overview of Intervention and Emotion Regulation Measures

| Study | PP | Intervention | Control group | ER measure |
| --- | --- | --- | --- | --- |
| Slee et al., 2008 | BPD | **Cognitive Behavioural Therapy:** 10 weekly sessions developed for preventing self-harm (SH).Targets self-harm maintenance factors, including dysfunctional cognition, emotion regulation and problem solving. Emotion regulation targeted by mindfulness, acceptance and exposure with response prevention. (Standardized manual is available from authors) | **Treatment as usual:** Any treatment warranted. Most interventions involved limited number of individual CBT or inter-personal psychotherapy sessions. Social skills training was common. Authors did not record specific types of therapy or medication. | **Difficulties with Emotion Regulation Scale (DERS):** contains 6 scales of ER difficulties including (a) lack of awareness of emotional responses (e.g., I pay attention to how feel= reverse), (b) lack of clarity of emotional responses (e.g., I have difficulty making sense out of my feelings), (c) non-acceptance of emotional responses (e.g., When I’m upset, I feel ashamed with myself for feeling this way), (d) limited access to ER strategies (e.g., When I’m upset, I believe that there is nothing I can do to make myself feel better), (e) difficulties controlling impulses (e.g., When I’m upset, I feel out of control), and (f) difficulties engaging in goal-directed behaviours when experiencing negative emotions (e.g., When I’m upset, I have difficulty concentrating). All questions are self-rated from 1 (almost) never to 5 (almost) always. Scores on subscales range from 5–25 (Clarity, Goals), from 6–30 (Awareness, Non-acceptance) and from 7–35 (Impulses, Strategies). All of the subscales have adequate internal consistency, with alpha reliabilities of 80 or higher.  (See: *Gratz, K.L., & Roemer, L. (2004). Multidimensional assessment of emotion regulation and dysregulation: development, factor structure, and initial validation of the difficulties in emotion regulation scale. Journal of Psychopathology and Behavioural Assessment, 26, 41– 54.)* |
| Schuppert et al., 2012 | BPD | **Emotion Regulation Training:** Improve feeling of control over intense, strong emotions by increasing cognitive, social, and behaviour coping skills. ERT is based on CBT and DBT. 17 weekly sessions (a 105min).Consists of 3 stages. Stage 1: psychoeducation on emotion dysregulation, automatic thoughts and cognitive-behavioural chain analysis, skills. Phase 2: Improve locus of control and insight regarding personal strengths and pitfalls. Phase 3: learning ER skills (e.g., taking distance, or challenging negative, distorted assumptions), followed by attention to lifestyle. | **Treatment as usual:** included medication,  psychotherapy, inpatient psychiatric care and emergency services in case of self-harm or suicidal behaviour. | **Life Problems Inventory (LPI**) with 15item **emotion dysregulation** **subscale.** LPI assesses core aspects of. Emotional Dysregulation scale measures high sensitivity, high reactivity of emotional responses, slow return to baseline mood, episodic depression and suicidal ideation, irritability, anxiety, and problems with anger and other emotions. A sample item is: “Once I get upset, it takes me a long time to calm down. Authors reported that no specific instruments were available to assess ER problems in youth.  (See: *Miller AL, Rathus JH, LinehanMM. Dialectical Behaviour Therapy with Suicidal Adolescents. New York: Guilford Press; 2007.)* |
| Suveg et al., 2017 | AD | **Emotion focused CBT:** 10 weekly, 1h sessions based on CBT (e.g., cognitive restructuring, relaxation, homework, exposure tasks) Specific content to address emotion regulation problems (e.g., guilt, sadness, anger, etc.). Sessions begin with discussion how therapist and child are feeling that day. Focus on how they know (e.g., behavioural and physiological cues) and why they feel that way. Therapist describes how doing or thinking can help themselves feel better. Therapist normalizes experience of emotion and models adaptive ER. Child is can practice ER by generating strategies to manage situations. In first sessions emotion is introduced for which causes and consequences are discussed. Last sessions, focus on practicing skills during exposure tasks. ECBT has exposure tasks for all emotional experiences. (Standardized manual is available from authors) | **Active control**: CBT based on Coping Cat protocol with 10 weekly 1h sessions. The first half of treatment focused on helping children learn skills to manage anxiety and the second half had children practice using their skills during exposure tasks. | **Emotion Regulation Checklist (ERC)** includes 24 items rated from never to always on a 4-point scale with 2 subscales. The **Emotion Regulation subscale (9 items)**, measures appropriate affective expressions, empathy, and emotional self-awareness (e.g., displays appropriate negative emotion in response to hostile, aggressive, or intrusive acts by peers, Can say when s/he is feeling sad, angry or mad, fearful, or afraid) The **Lability/ Negativity subscale** (15 items) assesses inflexibility, lability, and dysregulated negative affect (e.g., Responds angrily to limit setting by adults, Displays excessive energy or excitement that others find intrusive or disruptive).Higher scores indicate greater emotion regulation and greater emotion dysregulation, respectively. The ERC has been found to have substantial interrater reliability, discriminant validity and construct validity.  (See*: Shields, A, & Cicchetti, D. (1997). Emotion regulation among school-age children: The development and validation of a new criterion Q-sort scale. Developmental Psychology, 33, 906-917)*  **Children’s Emotion Management Scale (CEMS)** self-report measure to assess the ability to manage anger, sadness, and worry using a 3-point scale ranging from hardly ever to often. There are 3 subscales for each emotion: Inhibition, Dysregulated Expression, and Emotion Regulation Coping. Emotion Regulation Coping subscale serve as indices of adaptive emotion regulation. Inhibition and Dysregulated Expression subscales reflect maladaptive methods of managing emotions. The reliability and validity of CEMS have been established.  *(*See*: Zeman J, Cassano M, Suveg C, Shipman K (2010). Initial validation of the Children’s Worry Management Scale. Journal of Child and Family Studies,19,381-392.* and *Zeman J, Shipman K, Penza-Clyve S.(2001). Development and initial validation of the Children’s Sadness Management Scale. J Nonverbal Behav;25, 187-205)* |
| Dingle et al., 2017 | AD, MD | **Emotion regulation program** (TuneIn):  4 weekly 90min sessions. Helps young people identify, name, tolerate, and modify emotions strategically, using music as a tool. Each session participants are asked to a) draw an image that they associated with the music, b) indicate where they feel a physical response to music, and c) complete lyric analysis task to evoke and explore different emotional responses. | **Waitlist** | **Difficulties with Emotion Regulation Scale:**: See this Table: Slee et al., 2018 |
| Hides et al., 2011 | MD, SUB | **Cognitive Behavioural Therapy:** and motivational interviewing for 12 weeks. Authors did not provide detailed information on structure or content | **Treatment as usual:** Case management and motivational interviewing for substance misuse by social worker. | **Coping Inventory for Stressful Situations (CISS):** is a 48 item self-report measure that assesses 3 coping dimensions: task- (e.g., Think about how I solved similar problems), emotion- (e.g., Worry about what I am going to do) and avoidance oriented coping (e.g., watch TV, call a friend). The task-coping subscale has been shown to be negatively related to psychopathology and distress, while emotion-oriented coping was positively related to these constructs. CISS has been shown to have good validity and reliability  (See: *Endler &Parker, (1994).* *Assessment of multidimensional coping: task, emotion, and avoidance strategies*. *Psychological Assessment, 6, 50-60)* |
| Atkinson et al., 2011 | ED | Based on the **Mindfulness-based cognitive therapy**. Session 1: Identifying internal experiences relating to body and coping strategies. Thought suppression and magnification exercises demonstrate effects of avoidance and rumination. Guided raisin exercise and breathing. Session 2: Barriers to practising mindfulness. Guided decentring exercise. Session 3: Welcoming negative internal experiences. Negative body-related judgement and self-criticism. Suggestions for acceptance-based responses. Guided experiential exercise to practise acceptance. Daily homework: breathing, awareness and acceptance. | **Assessment only** | **Five Facet Mindfulness Questionnaire (FFMQ)** measures 5 key mindfulness constructs: Observing (‘I notice the smell and aroma of things’), describing (‘I am good at finding words to describe my feelings’), awareness (‘I find myself doing things without paying attention’-reverse), non-judging (‘I think some of my emotions are bad or inappropriate’-reverse) and non-reactivity (‘I perceive my feelings and emotions without having to react to them’). Participants rate 39 statements on a 5-point scale from never, very rarely, very often or always true. Higher scores reflect greater mindfulness. Good psychometric properties have been reported.  (See: *Baer R, Smith GT, Lykins E et al. Construct validity of the five facet mindfulness questionnaire in meditating and nonmeditating samples. Assessment 2008; 15: 329–42.)* |
| Azrin et al, 2011 | CD, SUB | **Individual** **Cognitive Problem-Solving**: based on problem-solving methods to improve self-control and problem-solving deficits. Purely cognitive: Use of the problem-solving steps reinforced (e.g., the youth was praised for generating or selecting a solution, but not for content or social wisdom of the solutions). Problem solving steps included: stop and think, state problem, brain storm solutions, consequences of solutions, pick best option. | **Active control:** Family behavioural therapy (FBT) addressing cognitive, verbal, social, and familial factors that are believed to influence drug use and antisocial behaviour. | **Social Problem-Solving Inventory-Revised** (SPSI-R) a 52-item self-report measure that yields five problem-solving summary scores (i.e., Negative Problem Orientation, Impulse Control Style, Avoidant Style, Positive Problem Orientation, and Rational Problem Style). Lower scores indicate better use of problem-solving strategies for the former three summary scales (NPO, ICS, AS), and higher scores indicate better problem-solving strategies for the latter two scales (PPO, RPS). The revised version was derived from the original SPSI)  (See: *D’Zurilla, T. J., Nezu, A. M., & Maydeu-Olivares, A. (in press). Manual for the Social Problem-Solving Inventory-Revised (SPSI-R). North Tonawanda, NY: Multi- Health Systems, Inc)* |
| Stasiak et al., 2014 | MD | **Computerized Cognitive Behavioural Therapy:** fantasy game. User selects avatar, different themes linked to particular content (e.g. cognitive restructuring techniques in Sky and Star Cities). Points for completion and rewarded at the end. Seven modules, each takes 25–30 minutes. Include mood monitoring, quiz, agenda setting, interactive exercises, animations and videos, summary of content and challenge. Paper Guidebook, with summaries, space to write down goals, answers and challenges (CBT education, behavioural activation, problem solving, cognitive restructuring, relaxation) | **Active control:** matched for module length, basic structure as cCBT but different content. (depression education, physical health, friends, time management, stress management, personal fulfilment) | **Adolescent Coping Scale** (short form) comprises 18 behaviours that adolescents use to deal with their concerns. Items are endorsed on a 5-point response scale (doesn’t apply or don’t do it; used very little; used sometimes; used often; used a great deal). It generates three subscales: Problem Solving, Reference to Others, and Non-Productive Coping. High scores on the Solving the Problem and Reference to Others subscales indicate positive coping strategies, whereas high scores on the Non-Productive Coping subscale indicate a less productive style of coping.  (See: *Frydeberg, E. and Lewis, R. (1993). Adolescent Coping Scale Admininistrator’s Manual. Melbourne: Australian Council for Educational Research (ACER))* |
| Jacobs et al., 2016 | MD | **Rumination-Focused Cognitive Behaviour** Therapy (RFCBT): 8 weekly 45-60min sessions that target rumination through psychoeducation and adopting functional analytic approach. Noticing rumination triggers, consequences and how to shift to more adaptive strategy such as mindfulness, behavioural activation, or problem-solving. Based on RFCBT for adults with adjustments. | **Assessment only** | **Ruminative response scale (RRS)** is a: 22-item self-report measure, with three subscales: reflection (‘Analyse recent events to try to understand why you are depressed’), brooding (‘What am I doing to deserve this?) and depression (‘Think about how alone you feel’) related. The RRS has been shown to be valid and reliable in young adolescent populations.  (See: *Treynor W, Gonzalez R, Nolen-Hoeksema S.(2003). Rumination reconsidered : A psychometric analysis. Cognitive Ther Res; 27: 247–259*.) |
| Livheim et al., 2015 | MD | **Acceptance Commitment Therapy**: Increase psychological flexibility: the ability to contact the present moment, based upon what the situation affords, to change or persist in behaviour in accordance with one’s values. ACT uses a unified model through six core processes: defusion, acceptance, flexible attention to the present moment, self-as-context, values, and committed action. These six core processes together make up the construct psychological flexibility. | **Treatment as usual** | **Avoidance and Fusion Questionnaire-Youth,** (AFQ-Y8) 8 items self-report measure to assess psychological inflexibility .Psychological inflexibility is seen in excessive cognitive fusion (‘the bad things I think about myself must be true’), and experiential avoidance. The AFQ-Y8 provides a single score with higher scores suggestive of greater inflexibility. Based on original 17 item measure,  (See: *Greco, L. A., Baer, R. A., & Lambert, W. (2008). Psychological inflexibility in childhood and adolescence: Development and evaluation of the Avoidance and Fusion Questionnaire for Youth. Psychological Assessment, 20(2), 93–102. doi:10.1037/ 1040-3590.20.2.93*)  **MAAS Mindful Attention Awareness Scale** is a 15-item scale designed to assess how attentive a person is of what is taking place in the present, with higher scores indicating higher levels of mindfulness. The scale has been validated in a number of studies and shows strong psychometric properties.  (See: *Brown, K. W., & Ryan, R. M. (2003). The benefits of being present: Mindfulness and its role in psychological well-being. Journal of Personality and Social Psychology, 84(4), 822–848*) |
| Kaufman et al., 2005 | MD, CD | **Cognitive Behavioural Therapy**: Adolescent Coping With Depression (CWD-A): group intervention that combines cognitive and behavioural strategies targeting problems that commonly characterize depressed youth. Behavioural skills precede cognitive skills. Intervention includes mood monitoring, increasing pleasant activities, social skills, and relaxation training | **Active control:**.Life Skill training (e.g., filling out a job application, renting an apartment), developed to fill a void in the upbringing of many at-risk youths. | Issues Checklist **Problem solving items**. Adolescents completed 18 potential conflict events from the Issues Checklist a measure of the number of parent–adolescent conflicts during the past 2 weeks and the average intensity of discussions regarding these issues (5-point scale ranging from 1 (calm) to 5(angry).  *(See: Clarke, G. N., Rohde, P., Lewinsohn, P. M., Hops, H., & Seeley, J. R. (1999). Cognitive–behavioral treatment of adolescent depression: Effi- cacy of acute group treatment and booster sessions. Journal of the American Academy of Child and Adolescent Psychiatry, 38, 272–279.)* |
| Hannesdottir et al., 2017 | ADHD | **Cognitive Behavioural Therapy:** OUtSMART focuses on teaching children social and emotional skills through cognitive-behavioural techniques and various executive function training components, including computerized working memory training. The latter has never been combined with CBT. | **Parent treatment program**: increase parenting self-esteem to reduce child’s ADHD symptoms. Parents learned about behaviour modification and environmental adjustment | **Emotion Regulation Checklist (ERC)** see this Table: Suveg et al. 2017 |
| Meisner-Stedman et al., 2017 | AD | **Cognitive therapy** for PTSD: 10 weekly sessions a 90 min. Treatment components included: psycho-education, activity scheduling/ reclaiming life, imaginal reliving, cognitive restructuring, re-visiting the site of trauma, stimulus discrimination with respect to traumatic reminders, direct work with nightmares, image transformation techniques and behavioural experiments The current programme did not include relaxation training or other arousal reduction techniques | **Waitlist** | **Trauma related rumination**: a 3 item self-report measure including: ’I keep wishing that I could go back in time and prevent the event from happening’, ‘Whenever I think of the event I wonder why it happened to us’ and ‘I am always wondering if my family or I might get hurt again.’ Participants can respond “never,” (1) “sometimes,”(2) “often,” (3) or “almost always” (4). The measure has been shown to have good internal consistency.  (See: *Meiser-Stedman, R., Shepperd, A., Glucksman, E., Dalgleish, T., Yule, W., & Smith, P. (2014). Thought control strategies and rumination in youth with acute stress disorder and post-traumatic stress disorder following single-event trauma. Journal of Child and Adolescent Psychopharmacol- ogy, 24,47–51)* |
| Latimer et al., 2003 | SUB | **Cognitive Behavioural Therapy:** Integrated Family and Cognitive-Behavioural Therapy. Modules (e.g. problem solving therapy) to foster cognitive skills (e.g. executive function-type skills). Three modules delivered to youth in a group format (i.e. Rational Emotive Therapy, Problem Solving Therapy, Learning Strategy Training) and one family therapy module (i.e. Problem-Focused Family Therapy) | **Active control**: Drugs Harm Psychoeducation curriculum (DHPE) by National Institute on Drug Abuse(see http:// www.nida.nih.gov/Infofax/Infofaxindex.html) | **Social Problem Solving Inventory** see this Table: Azrin et al., 2011 or  *D’Zurilla, T.J., Nezu, A.M., 1990. Development and preliminary evaluation of the social problem solving inventory. Psychol. Assess. 2, 156-163* |
| Winters et al.,2012 | SUB | **Motivational interviewing**: Session 1 focuses on eliciting information about alcohol and drug use and related consequences, assessing willingness to change. Examining pros and cons of use. Discussing goals. Session 2: progress in achieving goals, identifying high- risk situations, strategies to deal with social situations, willingness to change, negotiating long-term goals | **Waitlist** | **Social Problem-Solving Inventory**: see this Table: Azrin et al., 2011 or  *D’Zurilla, T.J., Nezu, A.M., 1990. Development and preliminary evaluation of the social problem solving inventory. Psychol. Assess. 2 (2), 156-163* |
| Smith et al., 2015 | MD | **Computerized Cognitive Behavioural Therapy**: Psycho education about depression and its treatment; behavioural activation; identifying and changing negative automatic thoughts; improving problem solving; improving social skills; relapse prevention. Treatment components are individually delivered via computer in an age-appropriate and appealing way, through the use of secure, interactive multimedia | **Waitlist** | **Child Response Styles Questionnaire** (CRSQ)-This 25-item questionnaire of ruminative thinking style has been shown to predict the severity and persistence of depression in adolescents.  (See: *Abela, J. R. Z., Rochon, A., & Vanderbilt, E. (2000). The children's response style questionnaire. Montreal, Canada: McGill University (Unpublished questionnaire)* |
| Fitzpatrick et al., 2005 | MD | **Problem Orientation** unit (35min video) of their skills manual: (a) increasing sensitivity to problems and to encourage an active coping model, (b) focusing attention on positive problem solving thoughts versus rumination and worry; (c) maximizing effort and persistence in the face of setbacks and emotional dis- tress; and (d) minimizing emotional distress while maximizing positive emotions. | **Active control:** Health Education video about general health | **Social Problem Solving Inventory-Revised** see this Table: Azrin et al., 2011 or *D’Zurilla, T.J., Nezu, A.M., 1990. Development and preliminary evaluation of the social problem solving inventory. Psychol. Assess. 2 (2), 156-163* |
| Hancock et al., 2016 | AD | **Acceptance and Commitment Therapy:** ProACTive based on ACT incorporating all six core therapeutic processes. Mindfulness practice each session. Psychoeducation of the ACT model . Values cards supported understanding of the concept of living a valued life. Defusion through experiential exercises. Graded exposure to enhance psychological flexibility. Emphasis was placed on mindful observation and acceptance of anxiety while faced with fear in order to foster committed action in line with self-identified values. Problem solving and social skills were incorporated. | **Waitlist** | **Avoidance and Fusion Questionnaire-Youth**, (AFQ-Y8) See this Table: Liveheim et al., 2015 |
| Afshari et al.,2014 | AD | **Emotion focused CBT**: facilitates the development of both emotion understanding and emotion regulation skills” 12 weekly sessions of approximately 1 h each. Therapists followed a treatment manual. The ECBT condition included core components of CBT (e.g., cognitive restructuring, relaxation, homework, exposure tasks). In ECBT children were engaged in different emotions other than anxiety. | **Waitlist** | **Cognitive Emotion Regulation Questionnaire (CERQ-k):** 36-items with 9 subscales: refocus on planning (positive strategy); rumination (negative strategy); putting into perspective (positive strategy); catastrophizing (negative strategy); positive refocusing (positive strategy); positive reappraisal (positive strategy); acceptance (positive strategy; self-blame (negative strategy); and other-blame (negative strategy). The higher the score, the more the strategy is employed.  (See: *Garnefski N, Rieffe C, Jellesma F, Terwogt MM, Kraaij V.(2007). Cognitive emotion regulation strategies and emotional problems in 9-11-year-old children: The development of an instrument. Eur Child Adolesc Psychiatry;16,1-9.)*  **Children’s Emotion Management Scale (CEMS)** see this Table: Suveg et al. 2017 |
